# Hepatic resection versus transarterial chemoembolization for the initial treatment of hepatocellular carcinoma: A systematic review and meta-analysis

**DOI:** 10.18632/oncotarget.4134

**Published:** 2015-05-14

**Authors:** Xingshun Qi, Diya Wang, Chunping Su, Hongyu Li, Xiaozhong Guo

**Affiliations:** ^1^ Department of Gastroenterology, General Hospital of Shenyang Military Area, Shenyang, China; ^2^ Department of Occupational and Environmental Health Sciences, Fourth Military Medical University, Xi'an, China; ^3^ Library of Fourth Military Medical University, Xi'an, China

**Keywords:** hepatocellular carcinoma, resection, chemoembolization, BCLC stage, survival

## Abstract

**Background & Aims:**

According to the Barcelona Clinic Liver Cancer (BCLC) staging system, hepatic resection and transarterial chemoembolization (TACE) should be recommended in patients with hepatocellular carcinoma (HCC) within and beyond the BCLC stage A, respectively. We conducted a systematic review and meta-analysis to compare the overall survival between HCC patients undergoing hepatic resection and TACE.

**Methods:**

PubMed, EMBASE, and Cochrane library databases were searched. All relevant studies were considered, if they reported the survival data in HCC patients undergoing hepatic resection and TACE. Hazard ratios (HRs) with 95% confidence intervals (CIs) were calculated for the comparison of cumulative overall survival. Odds ratios (ORs) with 95%CIs were calculated for the comparison of 1-, 3-, and 5-year survival rates. Subgroup analyses were performed according to the BCLC stages and portal vein tumor thrombus (PVTT). Sensitivity analyses were performed in moderate- and high-quality studies and in studies published after 2005.

**Results:**

Fifty of 2029 retrieved papers were included. One, 15, and 34 studies were of high-, moderate-, and low-quality, respectively. The overall meta-analysis demonstrated a statistically significantly higher overall survival in hepatic resection group than in TACE group (HR=0.60, 95%CI=0.55-0.66). Additionally, 1-, 3-, and 5-year survival rates were statistically significantly higher in hepatic resection group than in TACE group (OR=1.82, 95%CI=1.56-2.14; OR=3.09, 95%CI=2.60-3.67; OR=3.48, 95%CI=2.83-4.27). The subgroup meta-analyses confirmed the statistical significance in HCC within the BCLC stage A (HR=0.72, 95%CI=0.64-0.80), in HCC beyond the BCLC stage A (HR=0.60, 95%CI=0.51-0.69), in HCC within the BCLC stage B alone (HR=0.48, 95%CI=0.25-0.90), and in HCC with PVTT (HR=0.78, 95%CI=0.68-0.91). The statistical significance was also confirmed by sensitivity analyses in moderate- and high-quality studies (HR=0.62, 95%CI=0.53-0.71) and in studies published after 2005 (HR=0.59, 95%CI=0.53-0.66).

**Conclusions:**

Based on a systematic review and meta-analysis, hepatic resection may be considered in HCC beyond the BCLC stage A. However, given the limitations of study quality, more well-designed randomized controlled trials should be warranted to confirm these findings.

## INTRODUCTION

Nowadays, Barcelona Clinic Liver Cancer (BCLC) stage is the sole system approved by the European Association for Study of the Liver (EASL) and American Association for the Study of Liver Disease (AASLD) guidelines for the prognostic classification and treatment selection of hepatocellular carcinoma (HCC) [1-2]. According to this staging system, hepatic resection should be recommended in the BCLC stage 0 or A HCC with a single nodule (i.e., “the patients do not have liver cirrhosis or have liver cirrhosis but still have well preserved liver function, normal bilirubin and hepatic vein pressure gradient < 10 mmHg”), and transarterial chemoembolization (TACE) should be recommended in the BCLC stage B HCC (i.e., “the patients have large/multifocal HCC but without vascular invasion or extrahepatic spread”).

Recently, based on the real-world data, several large scale studies suggested that hepatic resection might be also appropriate in HCC cases beyond the BCLC stage A. First, Farinati et al. analyzed the treatment selection and prognosis of 405 HCC cases in the BCLC stage B who were enrolled between 1986 and 2008 by the Italian Liver Cancer group [3]. Only 40% of HCC cases in the BCLC stage B underwent TACE. However, TACE achieved a significantly shorter survival time than hepatic resection in such patients (median: 27 months versus 37 months). Second, Vitale et al. analyzed the outcomes of 2090 HCC cases in the different BCLC stages who were enrolled between 2000 and 2012 by the Italian Liver Cancer group [4]. In the BCLC stages 0, A, and B, the net survival benefit of hepatic resection over non-surgical treatments was statistically significant. Third, Roayaie et al. analyzed the survival of 8,656 cases diagnosed with HCC between 2005 and 2011 in BRIDGE study [5]. Hepatic resection not only achieved a significantly better survival than other treatments in ideal candidates for resection, but also achieved a significantly better survival than TACE in non-ideal candidates for resection.

Herein, we conducted a systematic review and meta-analysis of available literatures to clarify the survival benefits of hepatic resection over TACE in HCC patients.

## RESULTS

### Systematic review

**Figure 1:**
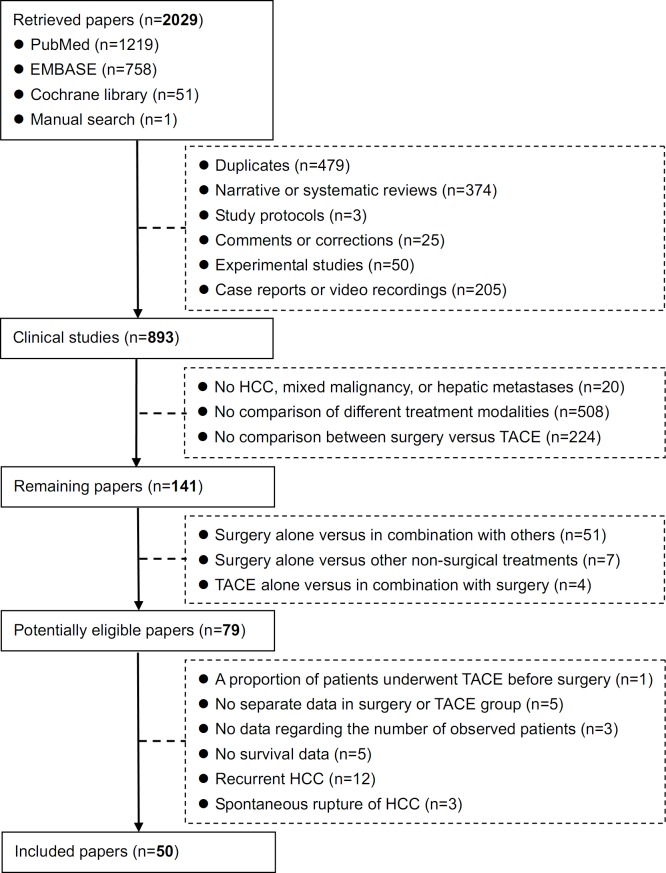
Flowchart of study inclusion

Overall, 2028 papers were initially retrieved via the three major databases, including 1219 papers in PubMed, 758 in EMBASE, and 51 in Cochrane library databases. One eligible paper was also manually identified. Among them, 50 papers were included in the systematic review (Figure 1) [6-55]. The study characteristics were summarized in Table 1. They were performed in Australia (n=1), Canada (n=1), China (n=18), Germany (n=7), India (n=1), Italy (n=5), Japan (n=6), Portugal (n=1), South Korea (n=8), Spain (n=1), and USA (n=1). The overall conclusions of every included study were summarized as follows: 1) the survival benefit of hepatic resection was statistically significant in 29 studies [7-8, 11, 16, 18-19, 23-24, 26, 28-29, 33-34, 36, 38, 40-41, 45-55]; 2) the survival was statistically similar between the two groups in 7 studies [12, 15, 20-22, 27, 30, 43]; and 3) the statistical difference was not reported in 14 studies [6, 9-10, 13-14, 17, 25, 31-32, 35, 37, 39, 42, 44]. The patients' characteristics and survival data of every study were shown in Supplementary Table 1. The eligibility criteria of every included study were shown in Supplementary Table 2. The criteria for treatment selection were shown in Supplementary Table 3. Only one study was of high quality, 15 studies were of moderate quality, and 34 studies were of low quality (Supplementary Table 4).

**Table 1:** Study characteristics: An overview of included studies

First author, Journal (Year)	Publication forms	Region	Study design	Period	Follow-up time	Target population	Survival benefits (statistical significance)
Cheng, Zhonghua Zhong Liu Za Zhi (2005)	Full text	China	Retrospective cohort study	2000.1-2003.1	NA	HCC with PVTT	NA.
Choi, World J Gastroenterol (2013)	Full text	South Korea	Retrospective cohort study	2003.1-2008.12	Median (range):38.6 (1-94) months in resection group	HCC 2-3 nodules, no vascular invasion, tumor diameter ≤5cm, Child-Pugh class A	Favor hepatic resection (significant).
Ciria, J Hepatol (2014)	Abstract	Spain	Retrospective cohort study	2006-2012	Median:20.5 months in all patients	BCLC stage B HCC	Favor hepatic resection (significant).
Colella, Transpl Int (1998)	Full text	Italy	Retrospective cohort study	1989.1-1997.6	Median:43 months in all patients	HCC without extrahepatic spread	NA.
El-Serag, J Hepatol (2006)	Full text	USA	Population-based study	1992-1999	NA	Medicare-enrolled patients with HCC in SEER registries (≥65 years old; <65 years old and disable or with end stage renal disease)	NA.
Fan, Eur J Surg Oncol (2014)	Full text	China Taiwan	Retrospective cohort study	2007.1-2012.12	Mean (range):19.5 (0-67) months in all patients	Aged ≥70 years, large HCCs ≥5 cm	Favor hepatic resection (significant).
Gerunda, Liver Transpl (2000)	Full text	Italy	Prospective cohort study	1988-1997.12	Mean:4.2±1.7 year in liver surgery group;NA in TACE group	Child-Pugh A or B; TNM stage I or II; <3-5cm or <3 nodules; no PVTT; no extrahepatic diseases	Statistically similar.
Guglielmi, HPB (2011)	Abstract	Italy	Retrospective cohort study	1991-2009	NA	HCC with cirrhosis	NA.
Guo, Ann Surg Oncol (2014)	Full text	China	Propensity score analysis	2003.3-2008.3	Before propensity-score matching: median: 35.0 months in resection group; 20.8 months in TACE group	BCLC stage A HCC, Child-Pugh class A	NA.
Hasse, Langenbecks Archiv für Chirurgie (1996)	Full text	Germany	Prospective cohort study	1990.1-1996.1	NA	Stage pT3 or pT4 HCC	Statistically similar.
Helmberger, Digestion (2007)	Full text	Germany	Retrospective cohort study	1995-2006	NA	HCC with VISUM stage 1	Favor hepatic resection (significant).
Herold, Liver (2002)	Full text	Germany	Retrospective cohort study	1988.1-1999.7	Mean (range):20 (0-119) months in all patients	Unselected HCC	NA.
Ho, Ann Surg Oncol (2009)	Full text	China Taiwan	Retrospective cohort study	1981.1-2000.6	Mean (range):20.2 (1-247.8) months	Multiple HCC	Favor hepatic resection (significant).
Hsu CY, Ann Surg Oncol (2012)	Full text	China Taiwan	Propensity score analysis	2002.1-2010.10	Mean: 18±16 months	HCC beyond the Milan criteria	Favor hepatic resection (significant).
Hsu KF, Eur J Radiol (2012)	Full text	China Taiwan	Retrospective cohort study	2001.1-2007.12	Mean:46.7±24.6 months in resection group;40.8±19.8 months in TACE group	Resectable early-stage HCC and Child-Pugh class A (BCLC stage A)	Statistically similar.
Huang, EJGH (1999)	Full text	China Taiwan	Cohort study	1984-1993	NA	Total: Resectable HCC, well-compensated liver function, tumor localized to a single lobe	Statistically similar.
						Subgroup: age >70 years	Statistically similar.
Jianyong, Medicine (2014)	Full text	China	Retrospective cohort study	2002.7-2008.11	NA	Total: BCLC stage B HCC	Statistically similar.
						Subgroup: 1 lesion of >5 cm in diameter	Favor hepatic resection (significant).
						Subgroup: 2-3 lesions (at least 1 lesion was >3 cm in diameter)	Favor hepatic resection (significant).
						Subgroup: >3 lesions of any diameter	Statistically similar.
Jin, J Gastrointest Surg (2014)	Full text	South Korea	Retrospective cohort study	1998.1-2013.4	Mean (range):18 (0.1-136) months	BCLC stage A HCC, solitary, large (>5 cm)	Favor hepatic resection (significant).
Kang, Hepatol Int (2010)	Abstract	South Korea	Retrospective cohort study	2003.1-2007.12	NA	Single HCC <3 cm	Favor hepatic resection (significant).
Kirchner, Transplant Int (2011)	Abstract	Germany	Retrospective cohort study	1993.3-2006.11	Mean:26.7±30.7 months	Unselected HCC	NA.
Lee JM, Hepatol Int (2014)	Abstract	South Korea	Cohort study	2000.1-2011.12	NA	HCC with PVTT	Favor hepatic resection (significant).
Lee YB, J Hepatol (2014)	Abstract	South Korea	Propensity score analysis	NA	NA	Resectable large solitary HCC	Statistically similar.
Lin, World J Surg (2010)	Full text	China Taiwan	Retrospective cohort study	2001.2-2007.12	NA	HCC, BCLC stage B, Child-Pugh A	Favor hepatic resection (significant).
Liu, Ann Surg Oncol (2014)	Full text	China Taiwan	Propensity score analysis	2002.2-2012.12	Mean:23±22months	HCC, BCLC stage C, PVTT	Favor hepatic resection (significant).
Luo, Radiology (2011)	Full text	China	Prospective cohort study	2004.1-2006.12	NA	Total: Large (≥5 cm), multiple, and resectable HCC	Statistically similar.
						Subgroup: 5-10 cm	Statistically similar.
						Subgroup: >10 cm	Statistically similar.
Markovic, J Hepatol (1998)	Full text	Italy	Prospective cohort study	1988.1-1993.12	Mean (range):40 (12-60) months	Unselected HCC (divided according to the Okuda stage and Child-Pugh class)	NA.
Martins, Liver Int (2006)	Full text	Portugal	Retrospective cohort study	1993.1-2003.12	Mean (range):66±11 (22-92) years	Unselected HCC	NA.
Min, JGH (2014)	Full text	South Korea	Propensity score analysis	2000-2009	Median (range):14.5 (0-103) months	Huge HCC (≥10 cm in diameter)	Favor hepatic resection (significant).
Nagashima, Int J Oncol (1999)	Full text	Japan	Retrospective cohort study	1989.1-1996.6	NA	Curatively unresectable intrahepatic multiple HCC with the main tumor ≥30 mm in size	Favor hepatic resection (significant).
Obed, Langenbecks Arch Surg (2008)	Full text	Germany	Retrospective cohort study	1995-2000	Median (range):200 (16-2054) days in TACE group;399 (11-2220) days in resection group	Unselected HCC (divided according to UICC stage)	NA.
Park, J Gastroenterol Hepatol (2008)	Full text	South Korea	Prospective cohort study	2000.11-2003.12	Median:14.4 months	Subgroup: Child-Pugh class A; modified UICC stage I or II	Favor hepatic resection (significant).
						Subgroup: Child-Pugh class A; modified UICC stage III	Favor hepatic resection (significant).
Paul, Oncology (2009)	Full text	India	Retrospective cohort study (1990-2000), prospective cohort study (2001-2005)	1990-2005	Mean (median):7.4±10.3 (3) months	Unselected HCC	NA.
Peng, Cancer (2012)	Full text	China	Retrospective case-control study	2002.12-2007.12	Mean (range):16.3±1.12 (2.0-83.0) months in resection group;12.1±0.56 (2.0-53.0) months in TACE group	Total: Resectable HCC with PVTT	Favor hepatic resection (significant).
						Subgroup: Resectable HCC with type I PVTT	Favor hepatic resection (significant).
						Subgroup: Resectable HCC with type II PVTT	Favor hepatic resection (significant).
						Subgroup: Resectable HCC with type III PVTT	Statistically similar.
						Subgroup: Resectable HCC with type IV PVTT	Statistically similar.
						Subgroup: Resectable HCC with PVTT; tumor size ≤5 cm	Statistically similar.
						Subgroup: Resectable HCC with PVTT; tumor size >5 cm	Favor hepatic resection (significant).
						Subgroup: Resectable HCC with PVTT; single tumor	Favor hepatic resection (significant).
						Subgroup: Resectable HCC with PVTT; multiple tumor	Statistically similar.
Perry, Liver Int (2007)	Full text	Australia	Prospective cohort study	Since 1998	Median:33 months for survivors;9 months for patients who died	Unselected HCC	NA.
Sako, Anticancer Research (2003)	Full text	Japan	Retrospective cohort study	1993.4-2001.10	Mean (range):4.2 (0.3-10.8) years in all patients	HCV-related, single, small HCC	Favor hepatic resection (significant).
Sasaki, J Hepatobiliary Pancreat Surg (1998)	Full text	Japan	Retrospective cohort study	1980.1-1994.4	NA	Total: unselected HCC	Favor hepatic resection (significant).
						Subgroup: Liver Cancer Study Group of Japan stage I HCC	Statistically similar.
						Subgroup: Liver Cancer Study Group of Japan stage II HCC	Favor hepatic resection (significant).
						Subgroup: Liver Cancer Study Group of Japan stage III HCC	Favor hepatic resection (significant).
Schumacher, Ann Hepatol (2010)	Full text	Canada	Retrospective cohort study	1996.1-2006.12	NA	Unselected HCC	NA.
Sotiropoulos, Dig Dis Sci (2009)	Full text	Germany	Retrospective cohort study	1998.4-2007.5	Median (range):15.3 (0.2-144) months	HCC with cirrhosis and no prior tumor treatments	Statistically similar.
Toro, BMC Surg (2014)	Full text	Italy	Retrospective cohort study	2002.1-2012.12	NA	HCC aged >18 years, Child-Pugh class A or B	NA.
Ueno, J Hepatobiliary Pancreat Surg (2002)	Full text	Japan	Prospective cohort study	1990.1-1998.10	NA	Total: HCC; Child class B and C cirrhosis without lymph node or distant metastasis	Favor hepatic resection (significant).
						Subgroup: Prognostic score = 0	Favor hepatic resection (significant).
						Subgroup: Prognostic score = 1-2	Favor hepatic resection (significant).
						Subgroup: Prognostic score = 3	Statistically similar.
Utsunomiya, Ann Surg (2014)	Full text	Japan	Prospective cohort study	2000.1-2005.12	Mean:1.9±1.6 years in resection group;1.5±1.4 years in TACE group	Non-HBV and non-HCV HCC	Favor hepatic resection (significant).
Wang, Academic Journal of Second Military Medical University (2012)	Full text	China	Propensity score analysis	2003-2011	NA	Early-stage HCC	Favor hepatic resection (significant).
Wang, Dig Liver Dis (2013)	Full text	China Taiwan	Retrospective cohort study	2003-2008	NA	BCLC stage C; naïve HCC; ECOG score ≤2 and Child-Pugh class A	Favor hepatic resection (significant).
Worns, Scand J Gastroenterol (2012)	Full text	Germany	Retrospective cohort study	1997.1-2009.12	NA	HCC in non-cirrhotic liver	Favor hepatic resection (significant).
Yamagiwa, J Gastroenterol Hepatol (2008)	Full text	Japan	Retrospective cohort study	1995.1-2004.12	Median:1008 days in resection group;609 days in TACE group	Unselected HCC	Favor hepatic resection (significant).
Yang, Radiology (2014)	Full text	South Korea	Retrospective cohort study*	2005.1-2006.12	NA	HCC ≤3 cm in diameter, no vascular invasion, single nodule	Favor hepatic resection (significant).
Ye, World J Gastroenterol (2014)	Full text	China	Retrospective cohort study	2007-2009	NA	HCC with PVTT	Favor hepatic resection (significant).
Yin, J Hepatol (2014)	Full text	China	Randomized controlled trial	2008.11-2010.9	Median (95%CI):33.3 (28.1-53.8) months in surgery group;13.5 (9.5-18.4) months in TACE group	Resectable multiple HCC outside of Milan criteria	Favor hepatic resection (significant).
Zhang, J Surg Res (2014)	Full text	China	Retrospective cohort study	2005.1-2010.3	Median (range):28 (3-84) months in all patients	Total: HCC; multiple tumors involving both lobes of the liver	Favor hepatic resection (significant).
						Subgroup: BCLC stage A	Favor hepatic resection (significant).
						Subgroup: BCLC stage B	Favor hepatic resection (significant).
						Subgroup: BCLC stage C	Favor hepatic resection (significant).
Zhong, Ann Surg (2014)	Full text	China	Propensity score analysis	2000.1-2007.12	Median (range):31.2 (1-120.3) months in the overall analysis^#^	BCLC stage B/C HCC	Favor hepatic resection (significant).

**Abbreviations:** BCLC, Barcelona Clinic Liver Cancer; HCC, hepatocellular carcinoma; NA, not available; PVTT, portal vein tumor thrombus; UICC, Union International Centre Cancer; VISUM, Vienna survival model.

Notes:

* data in propensity score analysis was not collected due to the absence of relevant data.

# the follow-up length was not available in the propensity score analysis.

### Meta-analysis

Four of 50 papers were not included in the meta-analysis, because they reported only the survival times, but not the survival rates or Kaplan-Meier curves [17, 25, 32, 37]. The remaining 46 papers were included in the meta-analysis.

### Overall meta-analysis

**Figure 2:**
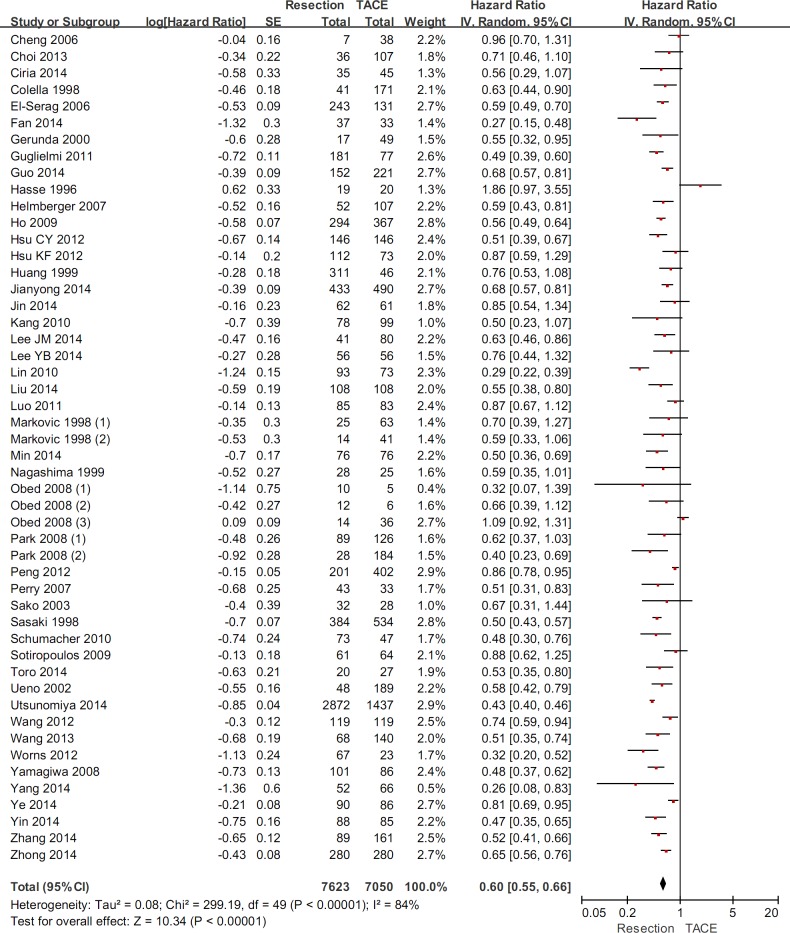
The overall meta-analysis comparing the overall survival between HCC patients undergoing hepatic resection and TACE

The overall meta-analysis demonstrated a statistically significantly higher overall survival in hepatic resection group than in TACE group (HR=0.60, 95%CI=0.55-0.66, P<0.00001) (Figure 2). The heterogeneity was statistically significant (P<0.00001; I^2^=84%). Funnel plots demonstrated that not all studies laid within 95%CI (Supplementary Figure 1).

Additionally, the meta-analyses demonstrated that 1-, 3-, and 5-year survival rates were statistically significantly higher in hepatic resection group than in TACE group (Table 2). There were statistically significant heterogeneities in all of the 3 meta-analyses.

**Table 2:** Comparisons of 1-, 3,- and 5-year survival between hepatic resection and TACE groups: Results of meta-analyses

Variables	No. included studies	Hepatic resection group		TACE group		Effect size		Heterogeneity	
		No. Pts. observed	No. Pts survival	No. Pts. observed	No. Pts survival	Odds ratio(95% CI)	P value	I^2^	P value
***All patients***									
1-year survival	41	6879	5689	6149	4279	1.82 (1.56-2.14)	<0.00001	50%	0.0001
3-year survival	41	7392	4778	6719	2542	3.09 (2.60-3.67)	<0.00001	70%	<0.00001
5-year survival	32	6551	3562	5710	1359	3.48 (2.83-4.27)	<0.00001	68%	<0.00001
***Within BCLC stage A***									
1-year survival	10	975	815	859	681	1.38 (1.00-1.91)	0.05	16%	0.3
3-year survival	10	975	586	859	398	1.92 (1.44-2.57)	<0.0001	36%	0.12
5-year survival	9	856	434	740	232	2.55 (1.61-4.06)	<0.0001	68%	0.002
***Beyond BCLC stage A***									
1-year survival	16	1827	1354	2346	1422	2.06 (1.57-2.71)	<0.00001	64%	0.0002
3-year survival	15	1789	940	2201	659	3.51 (2.45-5.02)	<0.00001	77%	<0.00001
5-year survival	8	1329	565	1723	379	2.89 (2.02-4.13)	<0.00001	66%	0.004
***BCLC stage B alone***									
1-year survival	3	561	474	608	472	2.38 (0.64-8.86)	0.2	91%	<0.0001
3-year survival	3	561	378	608	326	4.66 (1.01-21.5)	0.05	88%	0.0002
5-year survival	1	433	265	490	221	1.92 (1.48-2.50)	<0.00001	NA	NA
***PVTT***									
1-year survival	5	447	227	714	277	1.73 (1.17-2.57)	0.006	37%	0.17
3-year survival	4	440	129	676	90	2.72 (1.59-4.66)	0.0003	41%	0.17
5-year survival	2	309	86	510	40	7.34 (0.79-68.16)	0.08	88%	0.004
***Moderate- and high-quality studies***									
1-year survival	15	2097	1612	2369	1537	1.95 (1.50-2.52)	<0.00001	64%	0.0004
3-year survival	16	2134	1152	2402	792	3.04 (2.18-4.23)	<0.00001	78%	<0.00001
5-year survival	10	1631	732	1930	479	2.82 (1.99-4.00)	<0.00001	72%	0.0002
***Studies published after 2005***									
1-year survival	34	6385	5296	5688	3923	1.90 (1.61-2.23)	<0.00001	51%	0.0002
3-year survival	33	6492	4191	5573	2150	3.11 (2.58-3.74)	<0.00001	68%	<0.00001
5-year survival	25	5690	3166	4668	1161	3.62 (2.85-4.61)	<0.00001	71%	<0.00001

### Subgroup analysis in patients with different BCLC stages

**Figure 3:**
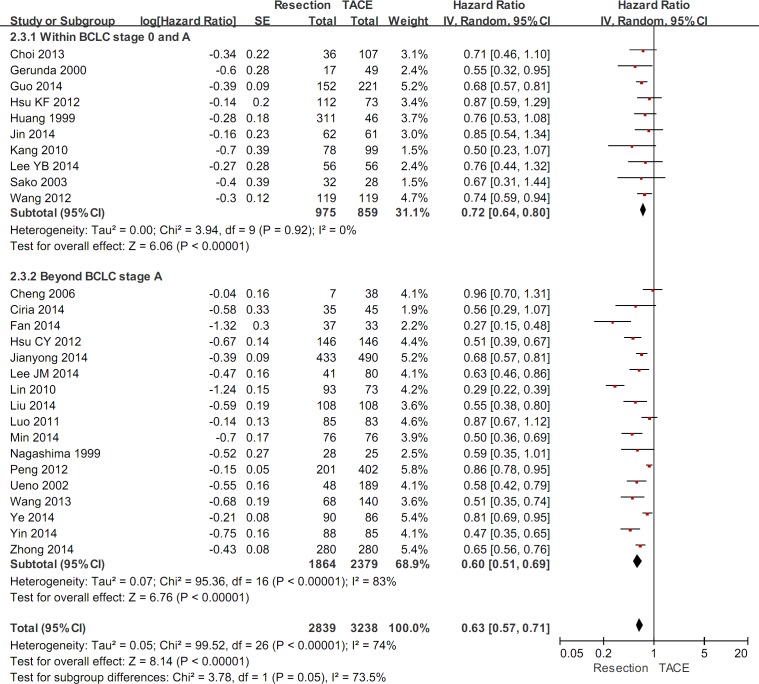
The subgroup meta-analysis comparing the overall survival between HCC patients within and beyond the BCLC stage A undergoing hepatic resection and TACE

In HCC patients within the BCLC stages 0 and A, the subgroup meta-analysis demonstrated a statistically significantly higher overall survival in hepatic resection group than in TACE group (HR=0.72, 95%CI=0.64-0.80, P<0.00001) (Figure 3). The heterogeneity was not statistically significant (P=0.92; I^2^=0%). Funnel plots demonstrated that all studies laid within 95%CI (Supplementary Figure 2).

Additionally, the meta-analyses demonstrated that 1-, 3-, and 5-year survival rates were statistically significantly higher in hepatic resection group than in TACE group (Table 2). There was a statistically significant heterogeneity in the meta-analysis of 5-year survival rate, but not in those of 1- and 3-year survival rates.

In HCC patients beyond the BCLC stage A, the subgroup meta-analysis demonstrated a statistically significantly higher overall survival in hepatic resection group than in TACE group (HR=0.60, 95%CI=0.51-0.69, P<0.00001) (Figure 3). The heterogeneity was statistically significant (P<0.00001; I^2^=83%). Funnel plots demonstrated that not all studies laid within 95%CI (Supplementary Figure 3).

Additionally, the meta-analyses demonstrated that 1-, 3-, and 5-year survival rates were statistically significantly higher in hepatic resection group than in TACE group (Table 2). There were statistically significant heterogeneities in all of the three meta-analyses.

There was a statistically significant subgroup difference (P<0.00001; I^2^=75%).

### Subgroup analysis in patients with BCLC stage B alone

**Figure 4:**
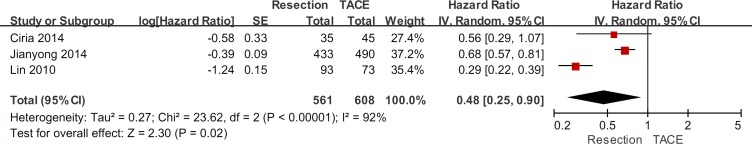
The subgroup meta-analysis comparing the overall survival between HCC patients with BCLC stage B alone undergoing hepatic resection and TACE

In HCC patients with BCLC stage B alone, the subgroup meta-analysis demonstrated a statistically significantly higher overall survival in hepatic resection group than in TACE group (HR=0.48, 95%CI=0.25-0.90, P=0.02) (Figure 4). The heterogeneity was statistically significant (P<0.00001; I^2^=92%). Funnel plots demonstrated that not all studies laid within 95%CI (Supplementary Figure 4).

Additionally, the meta-analyses demonstrated that 1-year survival rate was statistically similar between the two groups, but 3- and 5-year survival rates were statistically significantly higher in hepatic resection group than in TACE group (Table 2). There were statistically significant heterogeneities in the meta-analyses of 1- and 3-year survival rates. The heterogeneity could not be evaluated in the meta-analysis of 5-year survival rate.

### Subgroup analysis in patients with PVTT

**Figure 5:**
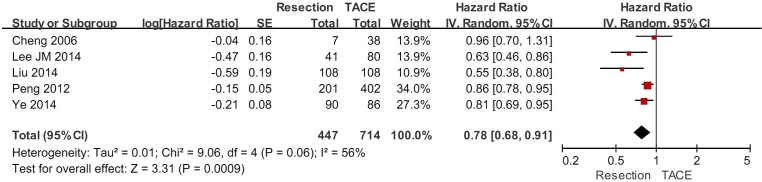
The subgroup meta-analysis comparing the overall survival between HCC patients with PVTT undergoing hepatic resection and TACE

In HCC patients with PVTT, the subgroup meta-analysis demonstrated a statistically significantly higher overall survival in hepatic resection group than in TACE group (HR=0.78, 95%CI=0.68-0.91, P=0.0009) (Figure 5). The heterogeneity was statistically significant (P=0.06; I^2^=56%). Funnel plots demonstrated that not all studies laid within 95%CI (Supplementary Figure 5).

Additionally, the meta-analyses demonstrated that 1- and 3-year survival rates were statistically significantly higher in hepatic resection group than in TACE group, but 5-year survival rate was statistically similar between the two groups (Table 2). There was a statistically significant heterogeneity in the meta-analysis of 5-year survival rate, but not in those of 1- and 3-year survival rates.

### Sensitivity analyses in moderate- and high-quality studies

**Figure 6:**
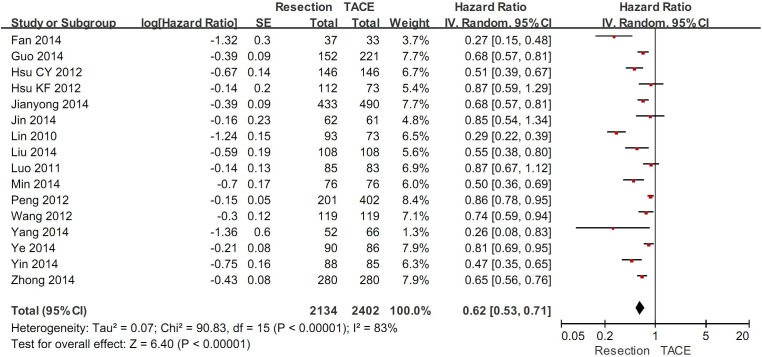
Sensitivity analysis in moderate- and high-quality studies

In 16 moderate- and high-quality studies, the sensitivity analysis demonstrated a statistically significantly higher overall survival in hepatic resection group than in TACE group (HR=0.62, 95%CI=0.53-0.71, P<0.00001) (Figure 6). The heterogeneity was statistically significant (P<0.00001; I^2^=83%). Funnel plots demonstrated that not all studies laid within 95%CI (Supplementary Figure 6).

Additionally, the meta-analyses demonstrated that 1-, 3-, and 5-year survival rates were statistically significantly higher in hepatic resection group than in TACE group (Table 2). There were statistically significant heterogeneities in all of the 3 meta-analyses.

### Sensitivity analyses in studies published after 2005

**Figure 7:**
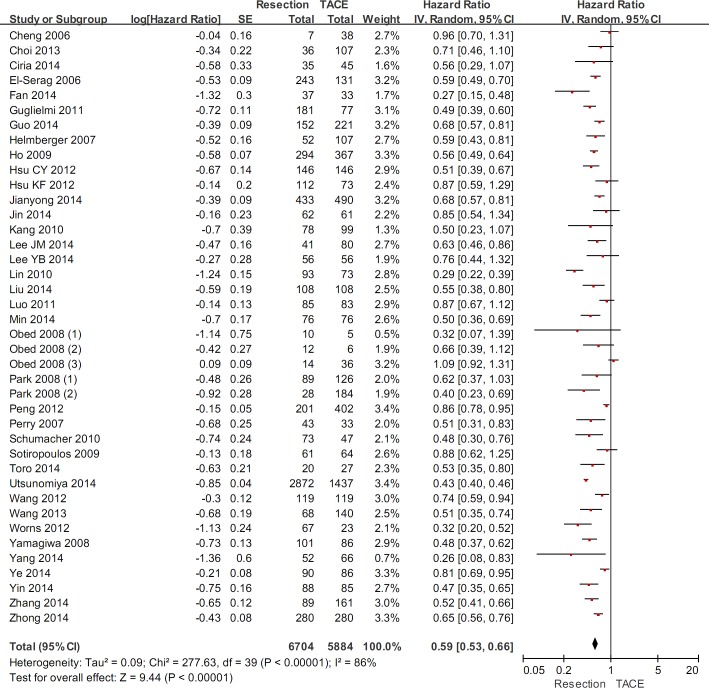
Sensitivity analysis in studies published after 2005

In 37 studies published after 2005, the sensitivity analysis demonstrated a statistically significantly higher overall survival in hepatic resection group than in TACE group (HR=0.59, 95%CI=0.53-0.66, P<0.00001) (Figure 7). The heterogeneity was statistically significant (P<0.00001; I^2^=86%). Funnel plots demonstrated that not all studies laid within 95%CI (Supplementary Figure 7).

Additionally, the meta-analyses demonstrated that 1-, 3-, and 5-year survival rates were statistically significantly higher in hepatic resection group than in TACE group (Table 2). There were statistically significant heterogeneities in all of the 3 meta-analyses.

## DISCUSSION

In theory, the BCLC staging system needs to be persistently updated with the dramatic improvement in the understanding of HCC and the invention of novel therapeutic modalities for HCC. Accumulated evidence suggests that the optimal treatment modality of HCC in the BCLC stage B may be further refined. In the present systematic review, we collected the comparative data regarding the overall survival in HCC patients undergoing hepatic resection and TACE. The overall meta-analysis demonstrated a statistically significant survival benefit of hepatic resection over TACE. In addition, considering the potential bias of patient selection, we performed several subgroup meta-analyses. All of them confirmed statistically significant survival benefits of hepatic resection over TACE.

At present, the curative treatment options of HCC mainly include liver transplantation (LT), hepatic resection, and radiofrequency ablation (RFA). Although LT is obviously superior to hepatic resection for the complete removal of tumor tissues, it is largely restricted by the scarcity of liver donors. On the other hand, hepatic resection appears to be superior to RFA for the improvement of overall survival and recurrence-free survival in HCC within the Milan criteria [56]. In clinical practices, hepatic resection is often regarded as the primary choice of therapy for early stage HCC, if the lesion is single, hepatic function is well-preserved, and portal hypertension is not severe. Under this circumstance, the results of our subgroup meta-analysis that hepatic resection could achieve a significantly better survival than TACE in HCC within the BCLC stage A were in line with our expectations.

The non-curative treatment options of HCC mainly include TACE and sorafenib. TACE is the first-line treatment option of HCC in the BCLC stage B. This recommendation is primarily attributed to the survival benefits of TACE over conservative or suboptimal treatments [57]. But we are not sure about whether TACE surpasses other active treatments for the improvement of overall survival. Our subgroup meta-analyses suggested that the survival was statistically significantly better in HCC patients beyond the BCLC stage A undergoing hepatic resection than in those undergoing TACE. Thus, hepatic resection might be also considered in selected HCC cases in the BCLC stage B.

After our study was registered, Kapitanov and colleagues published a similar meta-analysis to compare the short- and long-term results of hepatic resection versus TACE in HCC patients with cirrhosis [58]. They also concluded that the survival at 1 and 3 years was significantly better in patients treated with surgery than in those treated with TACE. Compared with their study, our systematic review and meta-analysis had several strengths. First, the selection of target population was broader and the number of included studies was larger. Thus, we were permitted to conduct more subgroup meta-analyses according to the study and patient characteristics. Second, the study quality was strictly assessed. Thus, we could readily understand the grade of current evidence. Third, the duplicate data were excluded, thereby avoiding the inflation of relevant information [59]. In the meta-analysis by Kapitanov et al., two papers by Zhong et al. were included. However, they reported the overlapping data. In the first paper, 392 HCC patients in the BCLC stage B were enrolled between January 2000 and November 2007 [60]; and in the second paper, 860 HCC patients in the BCLC stages B and C were enrolled between January 2000 and November 2007 [55]. By comparison, the first paper with a smaller sample size was excluded from our meta-analysis. Indeed, four other papers conducted by the same study team were also excluded from our meta-analysis [61-64].

The limitations of our meta-analysis should be clearly emphasized. First, only one included study was a randomized controlled trial. Additionally, a majority of included studies were of low quality. Certainly, we conducted a subgroup meta-analysis of moderate- and high-quality studies to confirm the reliability of our findings. Second, the heterogeneity among studies was statistically significant in all but one meta-analysis of HCC cases within the BCLC stage A. We employed a random-effect model to produce a relatively conservative estimate. Third, the publication bias was statistically significant in a majority of meta-analyses, despite three major English-language databases were searched. Fourth, the overall survival was the sole outcome observed in our study. Thus, we could not capture the other advantages or disadvantages of hepatic resection versus TACE. However, it should be noted that the overall survival was the most important endpoint to measure the therapeutic effectiveness in HCC [65]. By contrast, the time to recurrence, progression-free survival, and disease-free survival were the secondary endpoints. We could hardly translate the improvements in these secondary endpoints into the clinical practice recommendations.

In conclusions, hepatic resection might provide a better overall survival than TACE in HCC beyond the BCLC stage A. However, we should acknowledge that the current evidence is of low-quality. Considering that the drawbacks of study designs potentially lead to the selection biases, more well-designed randomized controlled trials should be warranted to compare the survival benefit of hepatic resection versus TACE in such patients.

## METERIALS AND METHODS

This work was registered with PROSPERO on December 19, 2014 (registration number: CRD42014015618).

### Search strategy

The PubMed, EMBASE, and Cochrane Library databases were searched. Search items were as follows: (“Hepatectomy” OR “Liver resection” OR “Hepatic resection” OR “Liver surgery” OR “Hepatic surgery”) AND (“TACE” OR “transarterial chemoembolization”) AND (“HCC” OR “hepatocellular carcinoma” OR “hepatic carcinoma”). The last search was performed on December 18, 2014. Relevant literatures were also manually searched.

### Study selection

Only clinical studies including more than 10 patients were considered in the systematic review. Accordingly, duplicate papers among databases, redundant publications, narrative or systematic reviews, study protocols, comments, experimental studies, and case reports were excluded. If two or more papers by the same study team had the overlapping data, only one paper with more adequate data and/or a longer enrollment period would be included.

The inclusion criteria should be as follows.

*Participants:* patients with HCC.
*Interventions:* hepatic resection and TACE as initial treatment modalities.
*Comparisons:* hepatic resection versus TACE.
*Outcomes:* overall survival.

The exclusion criteria should be as follows.

1. Non-HCC.
2. Hepatic metastases.
3. Mixed malignancies.
4. Non-comparative studies.
5. No comparison between hepatic resection versus TACE.
6. TACE before and after hepatic resection.
7. Comparison between hepatic resection versus TACE for recurrent HCC.
8. Comparison between hepatic resection versus TACE for spontaneous rupture of HCC.
9. No separate data in the hepatic resection or TACE group.
10. No detailed data regarding the survival rate in the hepatic resection or TACE group.
11. No detailed data regarding the number of observed patients in the hepatic resection or TACE group.

Notably, the major reason for exclusion of studies including patients with recurrent HCC and spontaneous rupture of HCC was the discrepancy in the treatment selection and outcomes among them.

### Data extraction

The following data were extracted: the first author, publication year, publication form, region, enrollment period, study design, study population, follow-up time, inclusion and exclusion criteria, number of HCC cases, treatment selection, survival rate, survival times, and Kaplan-Meier curve analysis with log-rank test. If the propensity score matching analysis was performed, we just collected the survival data after the propensity score matching analyses. If both survival rates and Kaplan-Meier curves were presented, only the survival rates would be collected. If only Kaplan-Meier curves were presented, we extracted the cumulative 1-, 3-, and 5-year survival rates by using the Distance Tool in the Measurements menu of Foxit PDF Reader software version 5.4.4.1023 (Foxit Cooperation, California, USA). This software was freely downloaded.

### Study quality

Because both retrospective/prospective observational studies and randomized controlled trials were included in the present systematic review, we could not employ a single scale to evaluate the quality of all included studies. More importantly, because our study was designed to compare the overall survival between patients undergoing hepatic resection and those undergoing TACE, the study quality assessment should primarily focus on the comparability of patient characteristics between the two groups. According to the Newcastle-Ottawa scale and major prognostic factors of HCC [66], we developed the following 9 questions that were more specific to the study quality assessment in the present systematic review.

1. Were the patients consecutively enrolled and prospectively followed?
2. Was the age statistically similar between the two groups?
3. Was the gender statistically similar between the two groups?
4. Was the Child-Pugh score/class or MELD score statistically similar between the two groups?
5. Were the diameter and number of tumor statistically similar between the two groups?
6. Was the BCLC stage or other HCC stage statistically similar between the two groups?
7. Were the criteria for treatment selection homogeneous between the two groups?
8. Was the follow-up time clearly reported?
9. Was the proportion of patients lost to follow-up less than 20%?

If the answers to 7-9 questions were “Yes”, the study would be considered to be of high quality. If the answers to 4-6 questions were “Yes”, the study would be considered to be of moderate quality. Otherwise, it would be considered to be of poor quality.

### Meta analysis

Only a minority of included studies clearly reported the hazard ratios (HRs) for the overall survival in HCC patients with hepatic resection versus TACE. Therefore, we calculated ln [HR] with standard error by using the calculation sheets which were developed by Matthew Sydes and Jayne Tierney [67]. The survival rates at different time points were entered into the calculation sheet of “(2a) curve data”. Accordingly, a curve was produced in the calculation sheet of “(2b) curve copy”, and ln[HR] and se(ln[HR]) could be available in the calculation sheet of “(4) output information”. Then, HRs with 95% confidence intervals (CIs) were pooled by using a random-effects model. Additionally, to provide the survival data in detail, we compared the 1-, 3-, and 5-year survival rates between HCC patients with hepatic resection versus TACE. The odd ratios (ORs) with 95% CIs were pooled by using a random random-effects model. In these meta-analyses, a P value of <0.05 was considered statistically significant. Heterogeneity between studies was assessed by using the I^2^ statistic (I^2^> 50% was considered as having substantial heterogeneity) and the Chi-square test (P<0.10 was considered to represent significant statistical heterogeneity). Funnel plots were performed to evaluate the publication bias. Subgroup meta-analyses were performed according to the BCLC stages (within versus beyond BCLC stage A). Subgroup difference between the two groups was evaluated by using the I^2^ statistic (I^2^> 50% was considered as having statistically significant difference) and the Chi-square test (P<0.10 was considered to represent statistically significant difference). Subgroup meta-analyses were also performed in patients with BCLC stage B alone and in those with portal vein tumor thrombus (PVTT). Sensitivity analyses were performed in moderate- and high-quality studies and studies published after 2005. All meta-analyses were conducted by using the statistical package Review Manager version 5.1.6 (Copenhagen, The Nordic Cochrane Center, The Cochrane Collaboration, 2011).

### SUPPLEMENTARY MATERIAL FIGURES AND TABLES










