# Supplementary material for: Hepatic resection versus transarterial chemoembolization for the initial treatment of hepatocellular carcinoma: A systematic review and meta-analysis
Source: Oncotarget. 2015 May 14;6(21):18715–33. doi: 10.18632/oncotarget.4134 (PMC4621923; doi:10.18632/oncotarget.4134)
Supplement: Supplementary file 1 [file oncotarget-06-18715-s001.pdf]

Hepatic resection versus transarterial chemoembolization for the initial treatment of hepatocellular carcinoma: A systematic review and meta-analysis

Supplementary Material

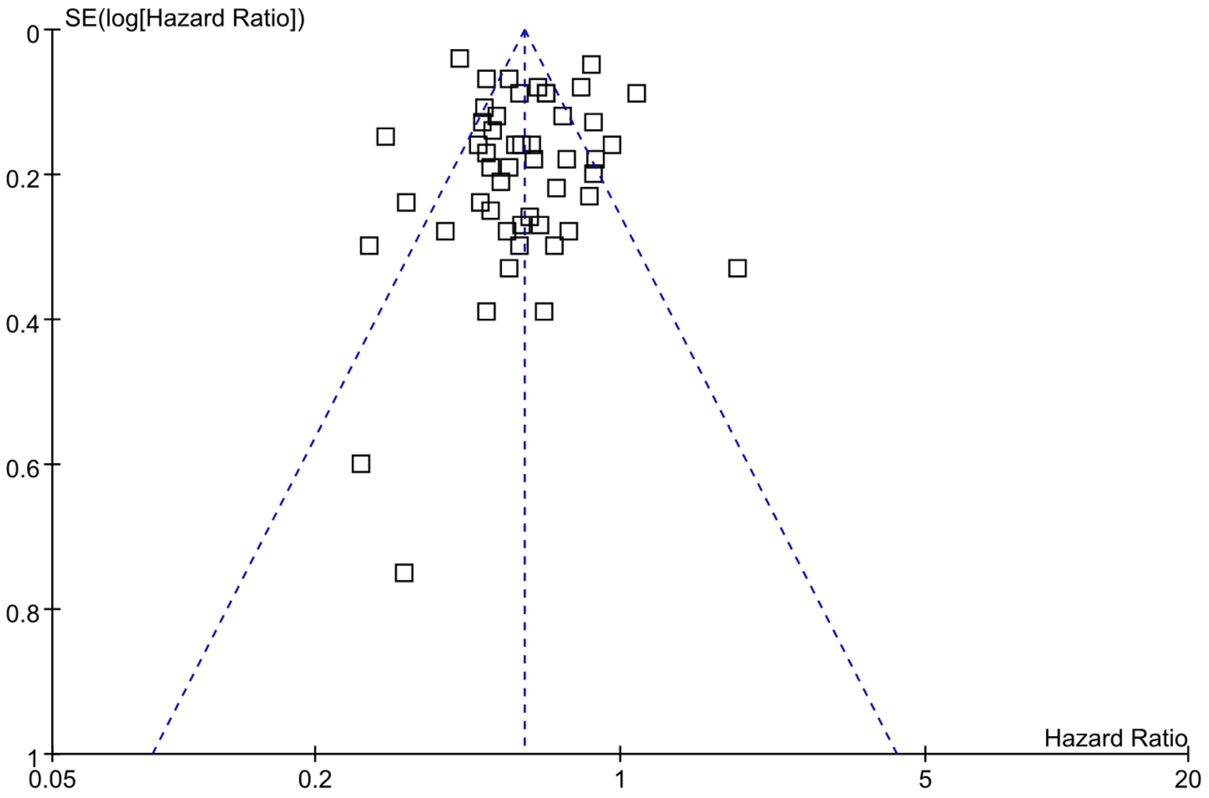

Supplementary Figure 1. Funnel plot to explore the publication bias in the overall meta-analyses.

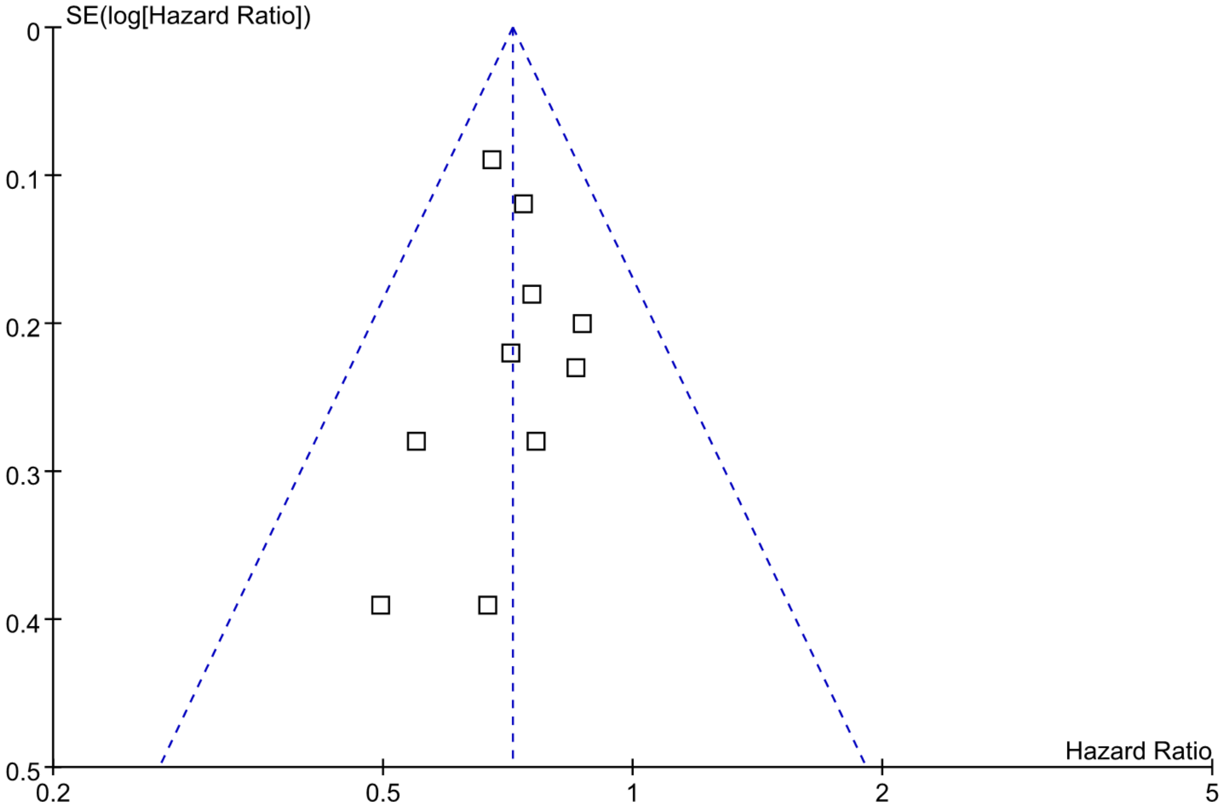

Supplementary Figure 2: Funnel plot to explore the publication bias in the meta-analysis of HCC patients within the BCLC stage A.

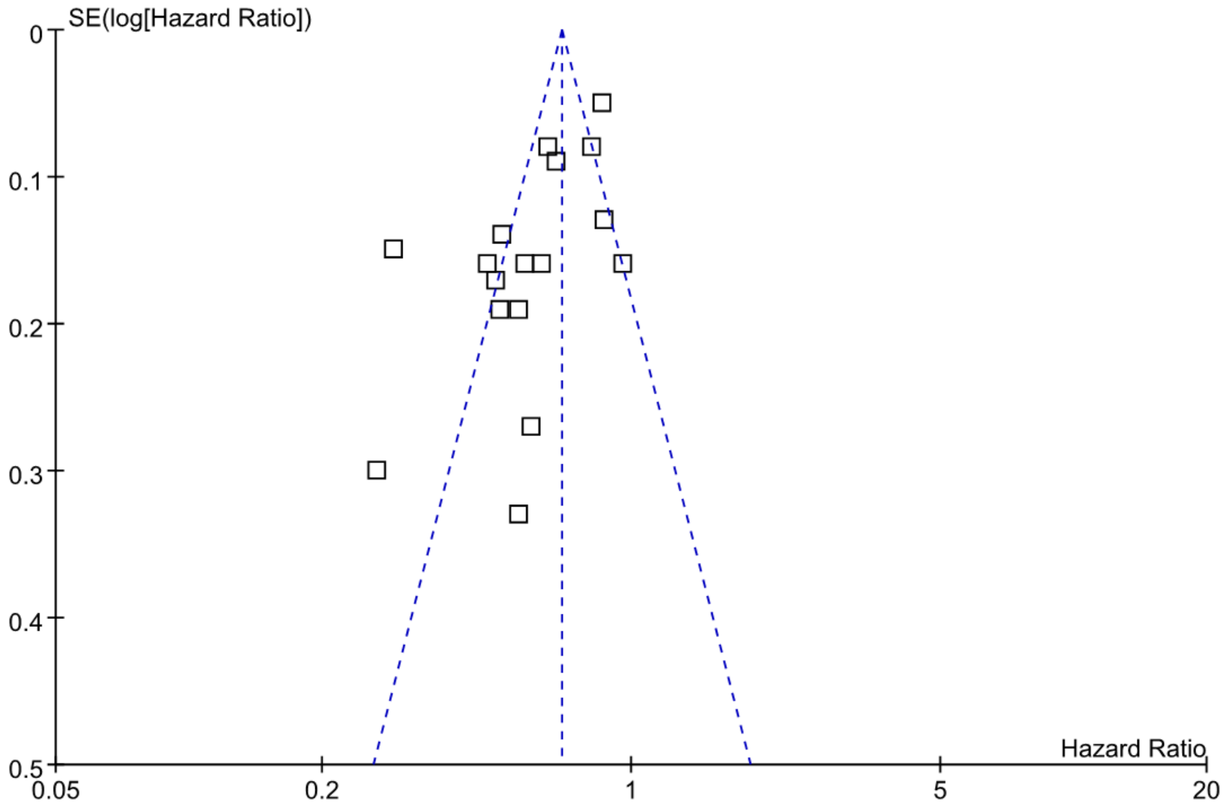

Supplementary Figure 3: Funnel plot to explore the publication bias in the meta-analysis of HCC patients beyond the BCLC stage A.

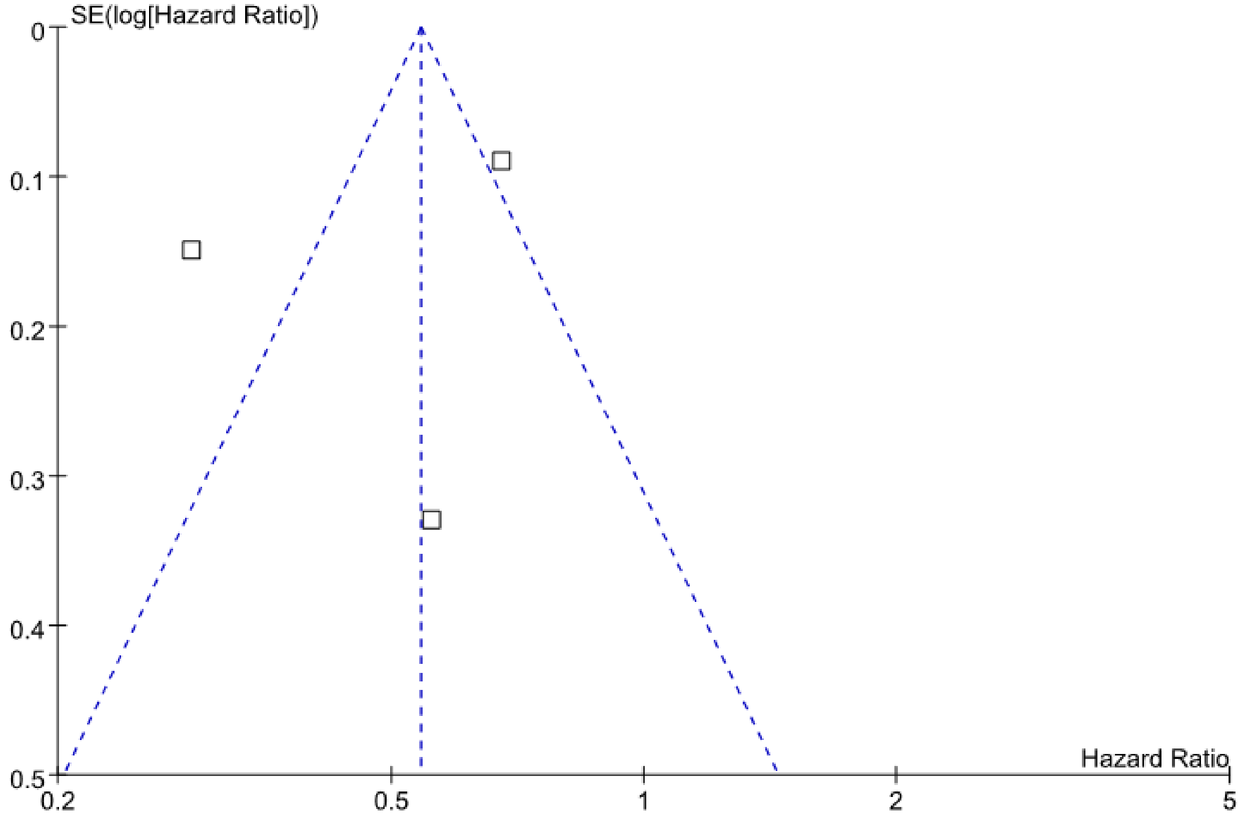

Supplementary Figure 4. Funnel plot to explore the publication bias in the meta-analysis of HCC patients with BCLC stage B alone.

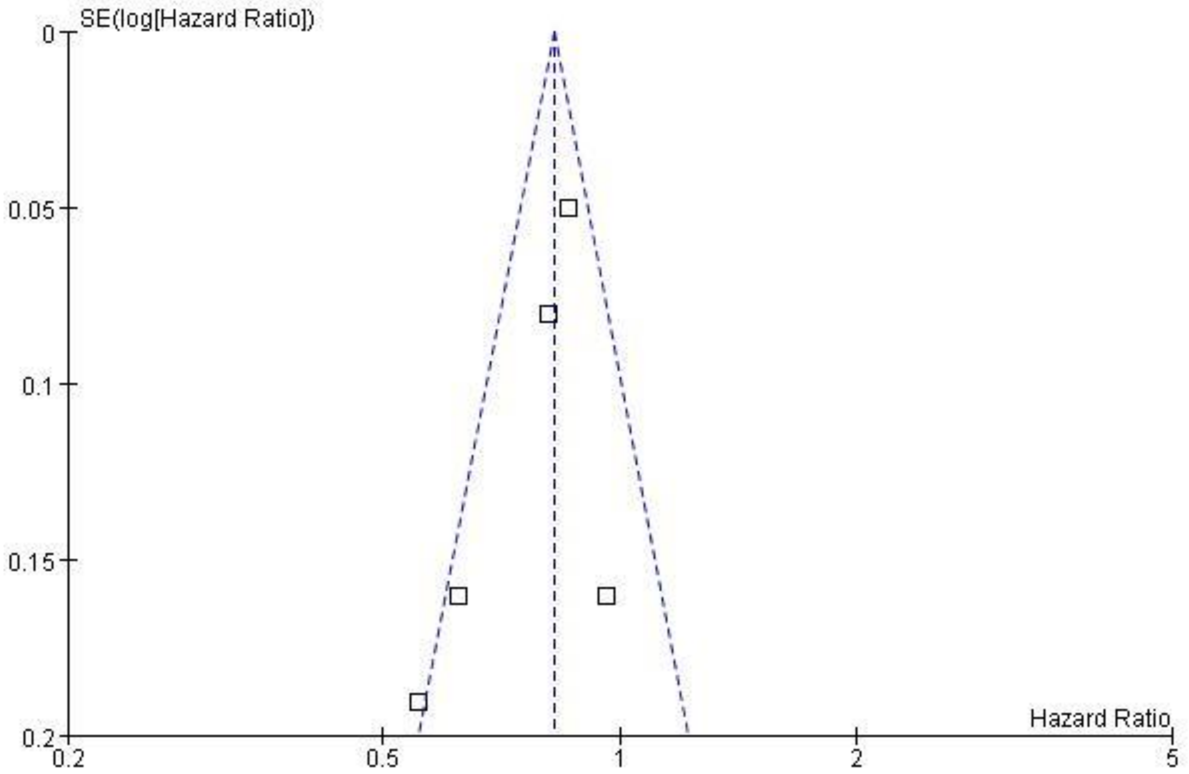

Supplementary Figure 5: Funnel plot to explore the publication bias in the meta-analysis of HCC patients with PVTT.

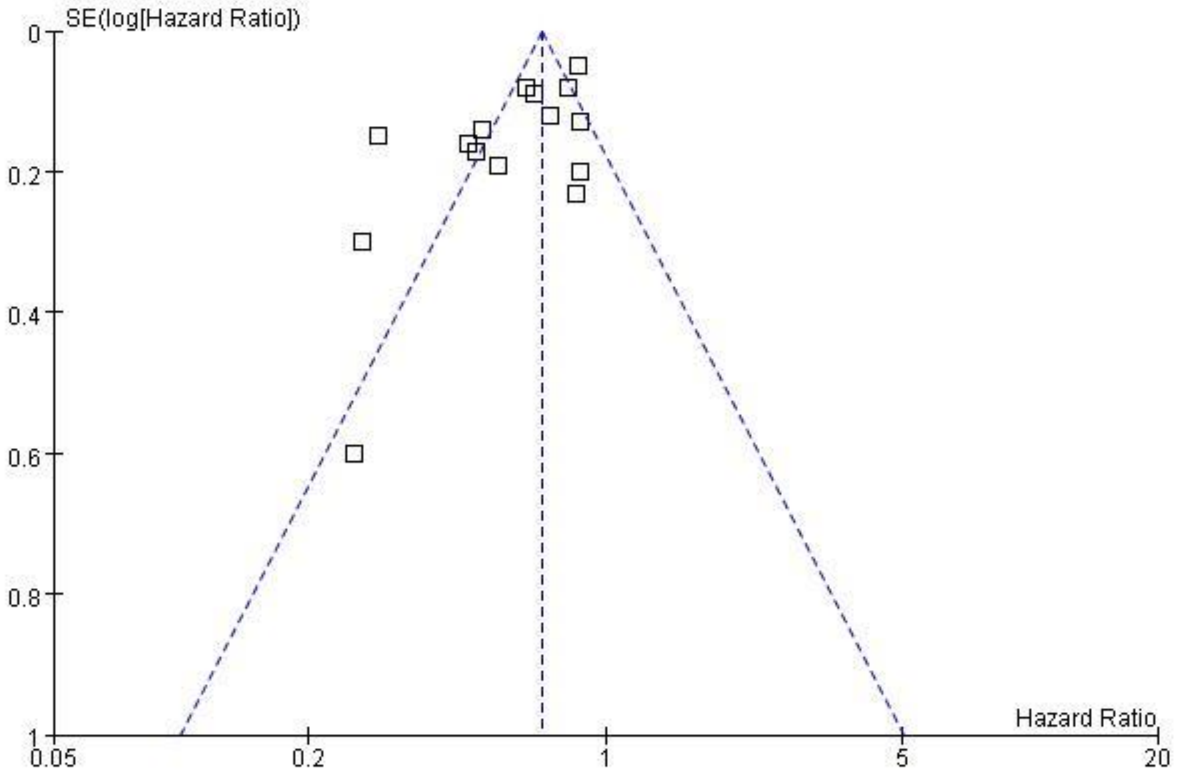

Supplementary Figure 6: Funnel plot to explore the publication bias in the meta-analysis of moderate- and high-quality studies.

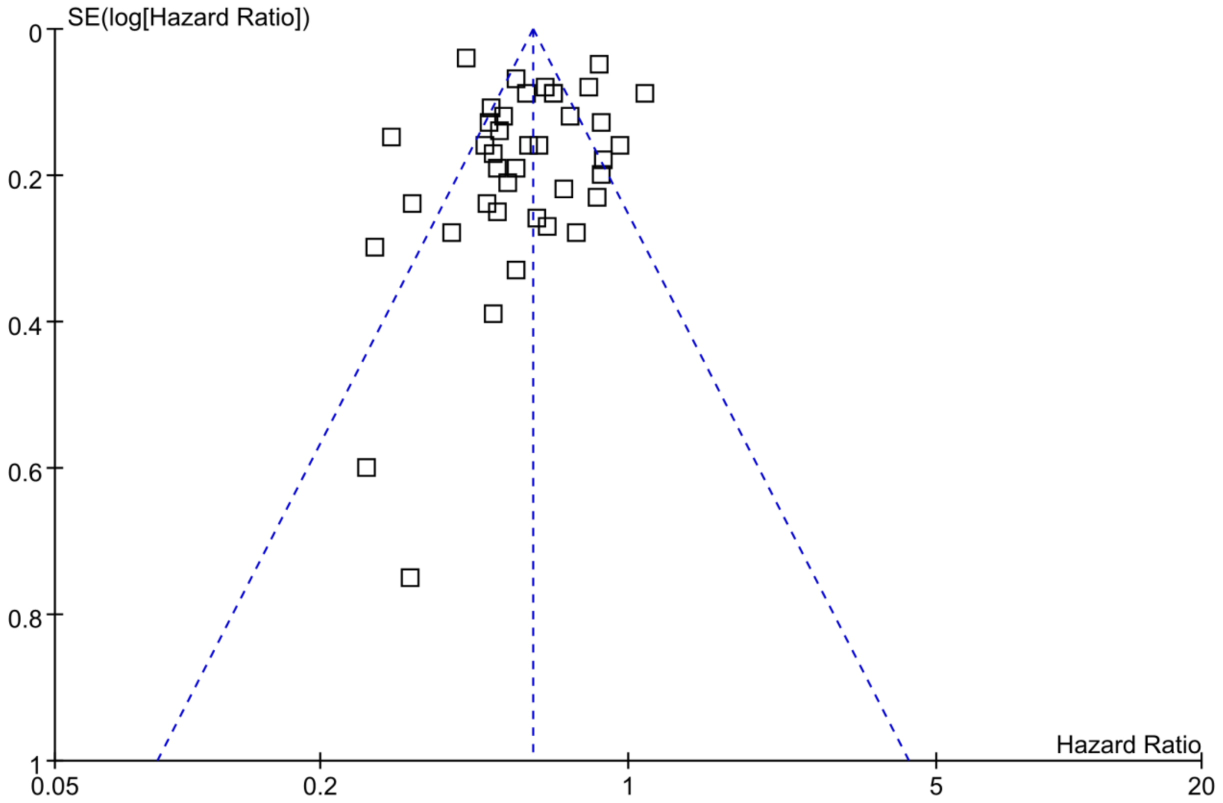

Supplementary Figure 7: Funnel plot to explore the publication bias in the meta-analysis of studies published after 2005.
