# Supplementary material for: Hepatic resection versus transarterial chemoembolization for the initial treatment of hepatocellular carcinoma: A systematic review and meta-analysis
Source: Oncotarget. 2015 May 14;6(21):18715–33. doi: 10.18632/oncotarget.4134 (PMC4621923; doi:10.18632/oncotarget.4134)
Supplement: Supplementary file 2 [file oncotarget-06-18715-s002.pdf]

Supplementary Table 1. Patients' characteristics and survival data: an overview of included studies

First author, Journal (Year)	Hepatic resection						TACE					
	No. Pts	Age (years)	Sex (Male/ female)	Underlying liver disease	No. liver cirrhosis	Survival data	No. Pts	Age (years)	Sex (Male/ female)	Underlying liver disease	No. liver cirrhosis	Survival data
Cheng, Zhonghua Zhong Liu Za Zhi (2005)	7	Mean±SD: 47.5±6.9	5/2	HBV (n=6)	NA	1-year overall survival rate: 14.3%; Median survival time (range): 8.0 (4.5-11.5) months	38	Mean±SD: 47.9±10.6	35/3	HBV (n=32)	NA	1-year overall survival rate: 10.5%; Median survival time (range): 5.0 (4.4-5.6) months
Choi, World J Gastroenterol (2013)	36	Mean±SD: 54.3±8.6	34/2	HBV (n=28)	20	1-, 3-, and 5-year overall survival rate: 91.7%, 83.3%, and 48.1%	107	Mean±SD: 61.2±9.3	86/21	HBV (n=67)	100	1-, 3-, and 5-year overall survival rate: 88.7%, 55.6%, 28.9%
Ciria, J Hepatol (2014)	35	NA	NA	NA	35	1-, 2-, and 3-year overall survival rate: 80%, 71.4%, and 68.6%	45	NA	NA	NA	45	1-, 2-, and 3-year overall survival rate: 71.1%, 55.6%, and 44.4%
Colella, Transpl Int (1998)	41	NA	NA	NA	NA	3- and 5-year overall survival rate: 64% and 44%	171	NA	NA	NA	NA	3- and 5-year overall survival rate: 32% and 22%
El-Serag, J Hepatol (2006)	243	<65 (n=16); 65-74 (n=139); >75 (n=88)	151/92	HBV (n=34); HCV (n=45); Alcohol (n=38)	NA	1-, 2-, and 3-year overall survival rate: 61.7%, 43.2%, and 30.9% Median survival time (25th-75th): 568 (200-1279) days	131	<65 (n=17); 65-74 (n=77); >75 (n=37)	91/40	HBV (n=30); HCV (n=45); Alcohol (n=43)	NA	1-, 2-, and 3-year overall survival rate: 44.3%, 18.3%, and 6.1% Median survival time (25th-75th): 324 (151-633) days

Fan,<br>Eur J Surg Oncol<br>(2014)	37	Median<br>(25th-75th):<br>76 (10.5)	28/9	HBV (n=14);<br>HCV (n=7);<br>HBV+HCV<br>(n=1);<br>No HBV or<br>HCV (n=15)	NA	3-year overall survival rate: 75.7%<br>Median survival time: 19.23<br>months	33	Median<br>(25th-75th):<br>77 (10.7)	21/12	HBV (n=12);<br>HCV (n=11);<br>No HBV or<br>HCV (n=10)	NA	3-year overall survival rate: 9.1%<br>Median survival time: 6.67<br>months
Gerunda,<br>Liver Transpl<br>(2000)	17	NA	NA	NA	NA	1-, 3-, and 5-year overall survival<br>rate: 71%, 53.2%, and 38%<br>Mean survival time: 4.3±0.9 years	49	NA	NA	NA	NA	1-, 3-, and 5-year overall survival<br>rate: 67.3%, 34.5%, and 0%
Guglielmi,<br>HPB<br>(2011)	181	NA	NA	NA	181	3- and 5-year overall survival rate:<br>57.7% and 44.9%	77	NA	NA	NA	77	3- and 5-year overall survival rate:<br>41.6% and 0%
Guo,<br>Ann Surg Oncol<br>(2014)	152	Mean±SD:<br>47.3±13.2	133/19	HBV (n=134)	NA	1-, 3-, and 5-year overall survival<br>rate: 75.5%, 44.8%, and 30.2%	221	Mean±SD:<br>48.1±12.6	139/13	HBV (n=132)	NA	1-, 3-, and 5-year overall survival<br>rate: 64.5%, 24.1%, and 13.7%
Hasse,<br>Langenbecks<br>Archiv für<br>Chirurgie<br>(1996)	19	Mean±SD:<br>62.9±7.6	1:2.8	HCV (47.4%)	36.8%	0.5-, 1-, 1.5-, and 2-year overall<br>survival rate: 42.1%, 31.6%,<br>31.6%, and 14.2%	20	Mean±SD:<br>63.1±11.3	1:2.5	HCV (36.8%)	45%	0.5-, 1-, 1.5-, and 2-year overall<br>survival rate: 72.3%, 50.1%,<br>41.2%, and 35.4%
Helmberger,<br>Digestion<br>(2007)	52	Mean±SD:<br>61.9±8.9	42/10	HCV (n=14);<br>HBV (n=6);<br>Alcohol<br>(n=11);<br>Other (n=21)	NA	1-, 3-, and 5-year overall survival<br>rate: 66%, 53%, and 30%<br>Median survival time (range): 37<br>(<1-96) months	107	Mean±SD:<br>64.6±10.0	89/18	HCV (n=22);<br>HBV (n=8);<br>Alcohol (n=28);<br>Other (n=41);<br>Mixed (n=8)	NA	1-, 3-, and 5-year overall survival<br>rate: 58.5%, 14%, and 4%<br>Median survival time (range): 13<br>(2-63) months

Herold, Liver (2002)	72	NA	NA	NA	72	Mean survival time: 31±5 months	31	NA	NA	NA	31	Mean survival time: 15±3 months
Ho, Ann Surg Oncol (2009)	294	Mean±SD: 57.0±11.8	240/54	HBV (n=200); HCV (n=82)	188	1-, 3-, and 5-year overall survival rate: 77.4%, 51.9%, and 36.6% Median survival time: 37.9 months	367	Mean±SD: 60.1±12.3	290/77	HBV (n=218); HCV (n=139)	270	1-, 3-, and 5-year overall survival rate: 62.6%, 25.2%, and 11% Median survival time: 17.3 months
Hsu CY, Ann Surg Oncol (2012)	146	<65 (n=66)	112/34	HBV (n=87); HCV (n=28); HBV+HCV (n=2); Alcohol (n=22)	NA	1-, 3-, and 5-year overall survival rate: 82%, 68%, and 46%	146	<65 (n=69)	111/35	HBV (n=87); HCV (n=28); HBV+HCV (n=2); Alcohol (n=22)	NA	1-, 3-, and 5-year overall survival rate: 65%, 29%, and 22%
Hsu KF, Eur J Radiol (2012)	112	Mean±SD: 60.3±11.7	76/36	HBV (n=62); HCV (n=36)	NA	1-, 3-, and 5-year overall survival rate: 93%, 71%, and 57% Median survival time: 46.8 months	73	Mean±SD: 61.9±11.7	39/34	HBV (n=37); HCV (n=32)	NA	1-, 3-, and 5-year overall survival rate: 91%, 66%, and 52% Median survival time: 37.9 months
Huang, EJGH (1999)	311	Mean±SD: 58±12	269/42	HBV (n=222)	206	1-, 2-, 3-, 4-, and 5-year overall survival rate: 78%, 66%, 55%, 48%, and 43%	46	Mean±SD: 62±11	40/6	HBV (n=31)	33	1-, 2-, 3-, 4-, and 5-year overall survival rate: 87%, 63%, 51%, 47%, and 34%
Jianyong, Medicine (2014)	433	Median (25th-75th): 53.0 (44.0-63.0)	315/118	HBV (n=388); HCV (n=8); Other (n=17); Negative (n=20)	NA	1-, 3-, and 5-year overall survival rate: 85.2%, 71.1%, and 61.2% Median survival time: 24.8 months	490	Median (25th-75th): 52.5 (43.0-63.0)	352/138	HBV (n=450); HCV (n=6); Other (n=12); Negative (n=20)	NA	1-, 3-, and 5-year overall survival rate: 84.1%, 62.2%, and 45.1% Median survival time: 26.9 months

Jin, J Gastrointest Surg (2014)	62	Median (range): 58 (29-79)	48/14	HBV (n=41); HCV (n=11); Alcohol (n=6); Others (n=4)	NA	1-, 3-, and 5-year overall survival rate: 83.2%, 75.7%, and 65%	61	Median (range): 59 (41-90)	53/8	HBV (n=35); HCV (n=4); Alcohol (n=11); Others (n=11)	NA	1-, 3-, and 5-year overall survival rate: 68.5%, 45%, and 17.5%
Kang, Hepatol Int (2010)	78	NA	NA	NA	NA	1-, 3-, and 5-year overall survival rate: 96%, 88.5%, and 88.5%	99	NA	NA	NA	NA	1-, 3-, and 5-year overall survival rate: 96.8%, 76.4%, and 61.6%
Kirchner, Transplant Int (2011)	102	NA	NA	NA	NA	Median survival time: 37 months	116	NA	NA	NA	NA	Median survival time: 16 months
Lee JM, Hepatol Int (2014)	41	NA	NA	NA	NA	1-, 2-, and 3-year overall survival rate: 63.6%, 31.3%, and 30% Median survival time: 19.9 months	80	NA	NA	NA	NA	1-, 2-, and 3-year overall survival rate: 36.3%, 9.8%, and 8.6% Median survival time: 6.6 months
Lee YB, J Hepatol (2014)	56	NA	NA	NA	NA	1-, 3-, and 5-year overall survival rate: 87.5%, 76.8%, and 64.3%	56	NA	NA	NA	NA	1-, 3-, and 5-year overall survival rate: 92.9%, 76.8%, and 53.6%
Lin, World J Surg (2010)	93	Mean±SD: 59.0±15.6	75/18	HBV (n=60); HCV (n=22)	NA	1-, 2-, and 3-year overall survival rate: 83%, 62%, and 49% Median survival time: 27.6 months	73	Mean±SD: 62.0±12.9	53/25	HBV (n=51); HCV (n=26)	NA	1-, 2-, and 3-year overall survival rate: 39%, 5%, and 2% Median survival time: 15.8 months

Liu,<br>Ann Surg Oncol<br>(2014)	108	Mean±SD:<br>62±15	91/17	HBV (n=50);<br>HCV (n=16);<br>HBV+HCV (n=2);<br>Alcohol (n=9);<br>Others (n=31)	NA	1-, 3-, and 5-year overall survival rate: 84%, 69%, and 59%<br>Median survival time: 64 months	108	Mean±SD:<br>62±14	84/24	HBV (n=49);<br>HCV (n=18);<br>HBV+HCV (n=4);<br>Alcohol (n=10);<br>Others (n=27)	NA	1-, 3-, and 5-year overall survival rate: 71%, 50%, and 35%<br>Median survival time: 32 months
Luo,<br>Radiology<br>(2011)	85	Mean±SD:<br>47.5±12.8	70/15	HBV (n=70);<br>HCV (n=2)	64	1-, 3-, and 5-year overall survival rate: 70.6%, 35.3%, and 23.9%<br>Median survival time (range): 22.5 (0.3-68.9) months	83	Mean±SD:<br>50.9±11.2	79/4	HBV (n=76);<br>HCV (n=4)	72	1-, 3-, and 5-year overall survival rate: 67.2%, 26%, and 18.9%<br>Median survival time (range): 19.5 (1.5-63.5) months
Markovic,<br>J Hepatol<br>(1998)	39	Mean (CI):<br>58 (53-62)	NA	NA	30	Median survival time (95%CI): 49 (23-74) months	116	Mean (CI):<br>60 (56-64)	NA	NA	106	Median survival time (95%CI): 31 (24-37) months
Martins,<br>Liver Int<br>(2006)	6	NA	NA	NA	NA	Median survival time: 40 months	49	NA	NA	NA	NA	Median survival time: 27 months
Min,<br>J Gastroenterol Hepatol<br>(2014)	76	Mean±SD:<br>53.0±12.0	63/7	HBV (n=57);<br>HCV (n=3);<br>No HBV or HCV (n=16)	NA	1-, 2-, and 3-year overall survival rate: 69.7%, 58.6%, and 51.7%	76	Mean±SD:<br>54.8±10.5	64/12	HBV (n=54);<br>HCV (n=2);<br>No HBV or HCV (n=20)	NA	1-, 2-, and 3-year overall survival rate: 40.2%, 33.9%, and 18.5%
Nagashima,<br>Int J Oncol<br>(1999)	28	Mean±SD:<br>61.0±8.0	21/7	HBV (n=6)	NA	1-, 3-, and 5-year overall survival rate: 78.6%, 48.7%, and 48.7%	25	Mean±SD:<br>63.0±9.0	20/5	HBV (n=1)	NA	1-, 3-, and 5-year overall survival rate: 64%, 22.9%, and 17.1%

Obed, Langenbecks Arch Surg (2008)	36	Mean (range): 52 (25-76)	26/10	NA	10	UICC stage I and II: 1- and 5-year overall survival rate: 100% and 80%; UICC stage III: 1- and 5-year overall survival rate: 63% and 80%; UICC stage IV a: 1- and 5-year overall survival rate: 38% and 0%	57	Mean (range): 59 (35-79)	41/6	NA	34	UICC stage I and II: 1- and 5-year overall survival rate: 72% and 27%; UICC stage III: 1- and 5-year overall survival rate: 62% and 0%; UICC stage IV a: 1- and 5-year overall survival rate: 38% and 4%
Park, J Gastroenterol Hepatol (2008)	117	NA	NA	NA	NA	Child-Pugh class A, modified UICC stage I or II: 1-, 2-, 3-, and 4-year overall survival rate: 93.3%, 87.6%, 83.9%, and 77.3%; Child-Pugh class A, modified UICC stage III: 1-, 2-, 3-, and 4-year overall survival rate: 85.7%, 75%, 63.1%, and 58.6%	409	NA	NA	NA	NA	Child-Pugh class A, modified UICC stage I or II: 1-, 2-, 3-, and 4-year overall survival rate: 92.1%, 84.9%, 71.7%, and 63.2%; Median survival time: 50.8 months Child-Pugh class A, modified UICC stage III: 1-, 2-, 3-, and 4-year overall survival rate: 72.3%, 47.3%, 28%, and 19.2% Median survival time: 22.0 months
Paul, Oncology (2009)	14	NA	NA	NA	NA	Median survival time (range): 19 (1-28) months	23	NA	NA	NA	NA	Median survival time (range): 11 (1-41) months

Peng, Cancer (2012)	201	Median (range): 55 (25-75)	187/14	HBV (n=172); HCV (n=4)	176	1-, 3-, and 5-year overall survival rate: 78.6%, 48.7%, and 48.7% Median survival time: 20.0 months	402	Median (range): 55 (23-75)	374/28	HBV (n=356); HCV (n=7)	363	1-, 3-, and 5-year overall survival rate: 37.8%, 7.3%, and 0.5% Median survival time: 13.1 months
Perry, Liver Int (2007)	43	NA	NA	NA	NA	1-, 2-, 3-, and 5-year overall survival rate: 76.9%, 69.8%, 51.8%, and 45.1%	33	NA	NA	NA	NA	1-, 2-, 3-, and 5-year overall survival rate: 76.9%, 49.4%, 29.8%, and 6.67%
Sako, Anticancer Research (2003)	32	NA	NA	HCV (n=32)	NA	1-, 3-, 5-, and 7-year overall survival rate: 100%, 79.4%, 70.1%, and 70.1%	28	NA	NA	HCV (n=28)	NA	1-, 3-, 5-, and 7-year overall survival rate: 92.9%, 65.9%, 52.8%, and 52.8%
Sasaki, J Hepatobiliary Pancreat Surg (1998)	384	Mean±SD: 60±8.4	3.7/1	NA	74%	3-, 5-, and 7-year overall survival rate: 77%, 48%, and 40%	534	Mean±SD: 62±7.4	4.7/1	NA	88%	3-, 5-, and 7-year overall survival rate: 36%, 19%, and 10%
Schumacher, Ann Hepatol (2010)	73	Mean±SD: 61±14	60/13	HBV (n=42); HCV (n=5); HBV+HCV (n=3); No HBV or HCV (n=23)	NA	1-, 2-, 3-, and 5-year overall survival rate: 85%, 76%, 71.5%, and 62.5% Mean survival time: 93 months	47	Mean±SD: 61±11	36/11	HBV (n=30); HCV (n=8); HBV+HCV (n=1); No HBV or HCV (n=8)	NA	1-, 2-, 3-, and 5-year overall survival rate: 73.5%, 62.5%, 45%, and 32% Mean survival time: 47 months
Sotiropoulos, Dig Dis Sci (2009)	61	NA	NA	NA	NA	1-, 2-, 3-, 4-, and 5-year overall survival rate: 51.66%, 47.83%, 43.17%, 32.03%, and 22.87% Median survival time (range): 11 (0.2-67.4) months	64	NA	NA	NA	NA	1-, 2-, 3-, 4-, and 5-year overall survival rate: 59.67%, 24.24%, 13.46%, 10.47%, and 10.47% Median survival time (range): 14 (1.2-143.9) months

Toro, BMC Surg (2014)	20	Mean±SD: 63.56±9.87	18/2	NA	NA	1-, 2-, 3-, and 5-year overall survival rate: 86.21%, 58.62%, 37.24%, and 16.55% Mean survival time (range): 31.8±28.57 (3-120) months	27	Mean±SD: 73±8.43	19/8	NA	NA	1-, 2-, 3-, and 5-year overall survival rate: 71.03%, 29.66%, 7.59%, and 0% Mean survival time (range): 17.2±12.17 (1-50) months
Ueno, J Hepatobiliary Pancreat Surg (2002)	48	Mean: 62.5	35/13	HBV (n=9); HCV (n=34); HBV+HCV (n=0); No HBV or HCV (n=3)	48	1-, 3-, and 5-year overall survival rate: 85.1%, 57.2%, and 37.1% Median survival time: 36.6 months	189	Mean: 62.7	134/55	HBV (n=32); HCV (n=132); HBV+HCV (n=1); No HBV or HCV (n=19)	189	1-, 3-, and 5-year overall survival rate: 77.9%, 27.2%, and 12.5% Median survival time: 15.3 months
Utsunomiya, Ann Surg (2014)	2872	Median (95%CI): 67 (50-79)	2332/540	Alcohol (n=874); HBV (n=0); HCV (n=0)	NA	1-, 3-, and 5-year overall survival rate: 91%, 77%, and 66%	1437	Median (95%CI): 69 (53-83)	1124/313	Alcohol (n=619); HBV (n=0); HCV (n=0)	NA	1-, 3-, and 5-year overall survival rate: 83%, 55%, and 32%
Wang, Academic Journal of Second Military Medical University (2012)	119	Mean±SD: 53.95±9.28	99/20	HBV (n=49)	NA	1-, 2-, 3-, and 4-year overall survival rate: 84.22%, 57.68%, 36.8%, and 24.09% Median survival time (95%CI): 2.5 (2.1-2.8) years	119	Mean±SD: 53.45±10.99	97/22	HBV (n=50)	NA	1-, 2-, 3-, and 4-year overall survival rate: 73.84%, 47.26%, 28.31%, and 10.69% Median survival time (95%CI): 2.5 (2.1-2.8) years

Wang, Dig Liver Dis (2013)	68	Mean±SD: 50.7±14.5	59/9	HBV (n=48); HCV (n=6)	NA	0.5-, 1-, and 2-year overall survival rate: 94.3%, 67%, and 59% Median survival time (95%CI): 33.4 (11.9-54.9) months	140	NA	NA	NA	NA	0.5-, 1-, and 2-year overall survival rate: 66.5%, 43.2%, and 21.4% Median survival time (95%CI): 9.2 (6.9-11.5) months
Worns, Scand J Gastroenterol (2012)	67	NA	NA	NA	NA	1-, 3-, and 5-year overall survival rate: 84%, 69%, and 42% Median survival time: 55.5 months	23	NA	NA	NA	NA	1-, 3-, and 5-year overall survival rate: 44%, 16%, and 11% Median survival time: 10.7 months
Yamagiwa, J Gastroenterol Hepatol (2008)	101	NA	NA	NA	NA	5-year overall survival rate: 58.9%	86	NA	NA	NA	NA	5-year overall survival rate: 14.7%
Yang, Radiology (2014)	52	Mean±SD: 55.7±10.6	38/14	HBV (n=36); HCV (n=6)	29	1-, 3-, and 5-year overall survival rate: 98%, 96%, and 93.6%	66	Mean±SD: 59.0±9.5	49/17	HBV (n=46); HCV (n=13)	60	1-, 3-, and 5-year overall survival rate: 96.9%, 82.9%, and 74.2%
Ye, World J Gastroenterol (2014)	90	Mean±SD: 49.3±10.7	81/9	HBV (n=12)	NA	1-, 2-, and 3-year overall survival rate: 28%, 20%, and 15% Mean survival time: 8.2 months	86	Mean±SD: 45.6±10.2	80/6	HBV (n=18)	NA	1-, 2-, and 3-year overall survival rate: 17.5%, 0%, and 0% Mean survival time: 7 months
Yin, J Hepatol (2014)	88	Mean±SD: 51.6±9.0	82/6	HBV (n=81); HCV (n=3)	69	1-, 2-, and 3-year overall survival rate: 76.1%, 63.5%, and 51.5% Median survival time (range): 41 (1-50) months	85	Mean±SD: 54.0±9.5	79/6	HBV (n=77); HCV (n=1)	74	1-, 2-, and 3-year overall survival rate: 51.8%, 34.8%, and 18.1% Median survival time (range): 14 (5-47) months

Zhang, J Surg Res (2014)	89	Mean±SD: 55.48±9.34	71/18	HBV (n=74); HCV (n=7)	18 *	1-, 3-, and 5-year overall survival rate: 78.9%, 49.4%, and 34.4% Median survival time: 34 months	161	Mean±SD: 57.24±10.58	134/27	HBV (n=146); HCV (n=9)	56 *	1-, 3-, and 5-year overall survival rate: 47.2%, 17.4%, and 8.6% Median survival time: 11 months
Zhong, Ann Surg (2014)	280	Median (range): 53 (19-78)	257/23	HBV (n=244); HCV (n=7)	NA	1-, 3-, and 5-year overall survival rate: 87%, 54%, and 34%	280	Median (range): 52 (19-82)	259/21	HBV (n=247); HCV (n=7)	NA	1-, 3-, and 5-year overall survival rate: 80%, 32%, and 15%

Abbreviations: CI, confidence interval; HBV, hepatitis B virus; HCV, hepatitis C virus; NA, not available; UICC, Union International Centre Cancer; SD, standard deviation;

Notes: * moderate or severe liver cirrhosis.
