# Supplementary material for: Hepatic resection versus transarterial chemoembolization for the initial treatment of hepatocellular carcinoma: A systematic review and meta-analysis
Source: Oncotarget. 2015 May 14;6(21):18715–33. doi: 10.18632/oncotarget.4134 (PMC4621923; doi:10.18632/oncotarget.4134)
Supplement: Supplementary file 3 [file oncotarget-06-18715-s003.pdf]

Supplementary Table 2. Patient selection: An overview of included studies

First author, Journal (Year)	Major inclusion criteria	Major exclusion criteria
Cheng, Zhonghua Zhong Liu Za Zhi (2005)	HCC with PVTT.	NA.
Choi, World J Gastroenterol (2013)	1) 2 or 3 radiologically diagnosed HCCs; 2) no radiologic vascular invasion; 3) Child-Pugh class A; 4) main tumor <5 cm in diameter; 5) platelet count >50,000/mm^3.	1) Small satellite nodules; 2) a tumor ≥ 5 cm in diameter; 3) macroscopic vascular involvement.
Ciria, J Hepatol (2014)	BCLC stage B HCC.	NA.
Colella, Transpl Int (1998)	HCC.	1) Incidental HCC at the time of liver transplantation and the fibrolamellar variant; 2) extrahepatic tumor spread at the time of treatment.
El-Serag, J Hepatol (2006)	1) All Medicare-enrolled patients with HCC in SEER registries; 2) persons ≥65 years old; 3) persons <65 years old are disabled or have end stage renal disease.	1) Stomach, colon, rectum, lung, pancreas, or breast cancers within the 5 years prior to the date of HCC diagnosis; 2) patients enrolled in a health maintenance organization during this time period.
Fan, Eur J Surg Oncol (2014)	1) Age >70 years; 2) HCC ≥5 cm.	1) Recurrent HCC; 2) distant metastases or lymph node involvement identified before treatment; 3) Child-Pugh class C liver cirrhosis.
Gerunda, Liver Transpl (2000)	1) Child-Pugh A or B; 2) TNM stage I or II; 3) < 3-5cm or <3 nodules; 4) no PVTT; 5) no extrahepatic diseases.	NA.
Guglielmi, HPB (2011)	Cirrhotic patients with HCC.	NA.
Guo, Ann Surg Oncol (2014)	Newly diagnosed BCLC stage A HCC.	NA.
Hasse, Langenbecks Archiv für Chirurgie (1996)	Stage pT3 or pT4 HCC.	NA.
Helmberger, Digestion (2007)	HCC patients who were divided into VISUM stage 1 (0-2 points), VISUM stage 2 (3 points), and VISUM stage 3 (4-6 points).	Patients with early HCC (single nodule <5 cm or 3 nodules <3 cm each) and impaired liver function who were considered for orthotopic liver transplantation.
Herold, Liver (2002)	All consecutive HCC patients admitted to the University of Erlangen-Nuernberg.	NA.
Ho, Ann Surg Oncol (2009)	Taiwanese patients with HCC.	Patients received their initial treatments for HCC at other hospitals.
Hsu, Ann Surg Oncol (2012)	HCC beyond the Milan criteria.	NA.
Hsu, Eur J Radiol (2012)	Resectable early-stage HCC (BCLC stage A).	1) Child-Pugh B liver function; 2) a subsequent liver transplantation; 3) a previous treatment history of radiofrequency ablation; 4) lost to follow-up.
Huang, EJGH (1999)	1) Resectable HCC; 2) well-compensated liver function; 3) tumor localized to a single lobe.	Severe liver diseases: gastrointestinal hemorrhage in the past month, clinical ascites, oedema, and encephalopathy.

Jianyong, Medicine (2014)	1) Intermediate HCC; 2) Child-Pugh A or B; 3) patients underwent liver resection or TACE; 4) BCLC stage B (lesion of >5cm in diameter or 2-3 lesions of which at least 1 was >3cm in diameter or >3 lesions of any diameter).	1) Other initial treatments for HCC; 2) extrahepatic metastasis; 3) diminished liver function (Child-Pugh C).
Jin, J Gastrointest Surg (2014)	1) Consecutive patients initially diagnosed as having a solitary large HCC; 2) BCLC stage A; 3) Child-Pugh class A or B; 4) curative surgery or TACE as an initial treatment.	1) Vascular invasion; 2) distant metastasis; 3) a history of previous treatment for HCC; 4) mixed cholangiocarcinoma; 5) refused treatment.
Kang, Hepatol Int (2010)	Single HCC <3 cm.	NA.
Kirchner, Transplant Int (2011)	HCC.	NA.
Lee, Hepatol Int (2014)	HCC with PVTT (segmental branch or extending to involve the right/left portal vein).	1) Main portal vein tumor thrombosis; 2) superior mesenteric vein tumor thrombosis; 3) Child-Pugh C.
Lee, J Hepatol (2014)	Resectable large solitary HCCs.	NA.
Lin, World J Surg (2010)	BCLC stage B, Child-Pugh class A, HCC.	1) Child-Pugh class B; 2) downstage with subsequent liver transplantation; 3) multimodality therapy (including cyberknife or radiofrequency ablation treatment); 4) distant metastasis; 5) lost to follow-up.
Liu, Ann Surg Oncol (2014)	HCC, BCLC stage C, PVTT	NA.
Luo, Radiology (2011)	1) Age: 18-75 years; 2) multiple nodules and main tumor ≥5 cm; 3) HCC with no previous treatment; 4) ECOG 0; 5) resectable disease, which was defined as the possibility of completely removing all tumors and retaining a sufficient liver remnant to maintain liver function, as assessed by our surgery team.	1) Vascular invasion or extrahepatic spread at imaging; 2) Child-Pugh class C liver cirrhosis or evidence of hepatic decompensation including ascites, esophageal or gastric variceal bleeding, or hepatic encephalopathy; 3) an American Society of Anesthesiologists score ≥3.
Markovic, J Hepatol (1998)	HCC was divided into Okuda stage 1-3 and Child-Pugh A-C	NA.
Martins, Liver Int (2006)	Consecutive patients with HCC, diagnosed between January 1993 and December 2003.	NA.
Min, JGH (2014)	Huge HCC (≥10 cm in diameter).	Any metastasis at the time of diagnosis.
Nagashima, Int J Oncol (1999)	Curatively unresectable intrahepatic multiple HCC with the main tumor ≥30 mm in size.	Distal metastasis before treatment
Obed, Langenbecks Arch Surg (2008)	Unselected HCC.	NA.
Park, J Gastroenterol Hepatol (2008)	Unselected HCC.	1) Patients were not treated at our hospital; 2) patients only required a second opinion.
Paul, Oncology (2009)	Unselected HCC.	NA.
Peng, Cancer (2012)	1) Age: 18-75 years; 2) HCC with no previous treatment; 3) PVTT on imaging; 4) ECOG 0; 5) resectable disease, which was defined as the possibility of completely removing all gross tumors and retaining a sufficient liver remnant to sustain life, as assessed by our surgery team.	1) Extrahepatic spread on imaging; 2) a Child-Pugh class C liver cirrhosis, ICG-R15 >30%, or evidence of hepatic decompensation including ascites, esophageal, or gastric variceal bleeding or hepatic encephalopathy; 3) an American Society of Anesthesiologists score ≥3.

Perry, Liver Int (2007)	Unselected HCC.	1) A follow-up of <12 months; 2) incomplete presentation data; 3) an initial presentation before 1997; 4) incidental tumours found at the time of transplantation.
Sako, Anticancer Research (2003)	HCV-related, single, small HCC.	1) Extrahepatic spread; 2) a tumor ≥3cm; 3) >1 tumor.
Sasaki, J Hepatobiliary Pancreat Surg (1998)	Unselected HCC.	NA.
Schumacher, Ann Hepatol (2010)	Unselected HCC (with complete data sets).	Patients with decompensated liver disease meeting the criteria for liver transplantation were referred to liver transplantation.
Sotiropoulos, Dig Dis Sci (2009)	1) HCC with cirrhosis; 2) no prior tumor treatments.	1) Extrahepatic liver disease; 2) operability/ operative risk; 3) prior tumor-specific treatments; 4) re-admission for HCC recurrence.
Toro, BMC Surg (2014)	1) age >18 years; 2) Child-Pugh class A or B; 3) HCC.	1) Child-Pugh class C; 2) patients with no indication for surgery refused to undergo radiofrequency ablation and TACE.
Ueno, J Hepatobiliary Pancreat Surg (2002)	1) Primary HCC; 2) Child-Pugh class B and C cirrhosis; 3) without lymph node or distant metastasis.	NA.
Utsunomiya, Ann Surg (2014)	HCC negative for both HBsAg and HCV Ab	1) Extrahepatic metastasis; 2) Child-Pugh C; 3) other treatments; 4) lacking outcome data.
Wang, Academic Journal of Second Military Medical University (2012)	Early HCC.	NA.
Wang, Dig Liver Dis (2013)	1) BCLC stage C; 2) naïve HCC; 3) ECOG score ≤2; 4) Child-Pugh class A.	1) Incomplete data; 2) Child-Pugh class B.
Worns, Scand J Gastroenterol (2012)	HCC in non-cirrhotic liver.	Cirrhosis.
Yamagiwa, J Gastroenterol Hepatol (2008)	Unselected HCC.	NA.
Yang, Radiology (2014)	A single-nodule HCC ≤3 cm without vascular invasion.	1) Child-Pugh class C liver function; 2) concomitant serious medical illness including malignant tumors other than HCC; 3) received multimodality treatment for the single-nodule HCC.

Ye, World J Gastroenterol (2014)	1) HCC with tumor thrombus in the first branch and/or main trunk of the portal vein, as confirmed by preoperative imaging or intraoperative exploration; 2) solitary tumor or multiple nodules that are mainly located in 1 or 2 adherent hepatic lobes and PVTT that can be removed with the tumors during preoperative assessment; 3) no distant metastasis; 4) no preoperative TACE/radiofrequency ablation; 5) candidates for hepatic resection and postoperative TACE with moderate hepatic function (Child-Pugh class A or B) and sufficient functional hepatic reserve; 6) candidates for TACE with moderate hepatic function (Child-Pugh class A or B) but insufficient hepatic functional reserve; 7) conservative therapy; 8) tumor size ≤10 cm.	NA.
Yin, J Hepatol (2014)	1) Good surgical risk patients 18-70 years; 2) ≥2 rounds of radiological imaging showing characteristic features of HCC, or one radiological imaging associated with alpha fetoprotein >400 µg/L, or cytological/histological evidence of HCC; 3) resectable HCC with tumors outside of Milan Criteria; 4) adequate liver remnant size after liver resection; 5) no radiological evidence of vascular invasion or extrahepatic metastasis; 6) tumors resectable with a single or multiple liver resections; 7) Child-Pugh A-B, with serum bilirubin ≤1.5 times the upper limit of normal, alanine aminotransferase and aspartate aminotransferase ≤2 times the upper limit of normal; 8) no major organ dysfunction; 9) hemoglobin ≥90g/L, white blood cell ≥3000cells/mm^3, platelets ≥80.000cells/mm^3; 10) informed consent had been obtained.	1) Cardiac, pulmonary, cerebral and renal dysfunction; 2) a history of other malignancy; 3) extrahepatic metastasis, portal vein or other major vascular involvement; 4) liver functional status of Child-Pugh C; 5) any other contraindication like: active gastrointestinal bleeding, refractory ascites, coagulopathy, severe portal hypertension; 6) no prior TACE or liver resection.
Zhang, J Surg Res (2014)	Multiple tumors (including satellitosis, multicentric tumors, intrahepatic metastases) involving both lobes of the liver metastasis.	NA.
Zhong, Ann Surg (2014)	Only Child-Pugh A patients with BCLC stage B/C HCC.	Metastasis to the lymph nodes and/or distant metastases.

Abbreviations: BCLC, Barcelona Clinic Liver Cancer; ECOG, Eastern Cooperative Group; HCC, hepatocellular carcinoma; NA, not available; PVTT, portal vein tumor thrombus; TACE, transarterial chemoembolization.
