# Supplementary material for: Hepatic resection versus transarterial chemoembolization for the initial treatment of hepatocellular carcinoma: A systematic review and meta-analysis
Source: Oncotarget. 2015 May 14;6(21):18715–33. doi: 10.18632/oncotarget.4134 (PMC4621923; doi:10.18632/oncotarget.4134)
Supplement: Supplementary file 4 [file oncotarget-06-18715-s004.pdf]

Supplementary Table 3. Treatment selection: An overview of included studies

First author, Journal (Year)	Criteria for hepatic resection	Criteria for TACE
Cheng, Zhonghua Zhong Liu Za Zhi (2005)	NA.	NA.
Choi, World J Gastroenterol (2013)	1) 2 or 3 radiologically diagnosed HCCs; 2) no radiologic vascular invasion; 3) main tumor <5 cm in diameter.	1) Tumor was ineligible for complete surgical removal; 2) low remnant liver volume was expected after resection; 3) patients declined operative intervention.
Ciria, J Hepatol (2014)	NA.	NA.
Colella, Transpl Int (1998)	No detailed criteria, but all patients who underwent hepatic resection had Child-Pugh A.	No detailed criteria, but all patients who underwent TACE had Child-Pugh A or B.
El-Serag, J Hepatol (2006)	NA.	NA.
Fan, Eur J Surg Oncol (2014)	1) Absence of prohibitive medical conditions; 2) favorable Child-Pugh class (A and selected B); 3) ability to resect all tumors while leaving an adequate volume of liver parenchyma (>30% of total liver volume); 4) absence of ascites; 5) total bilirubin level <2.0 mg/dL combined with an indocyanine green retention rate at 15 min of <20% or platelet count >100,000/uL.	1) Patients declined surgery; 2) bilirubin < 3 mg/dL and liver enzymes (aspartate aminotransferase and alanine aminotransferase) <270 IU/L; 3) no extra-hepatic metastasis; 4) no biliary obstruction; 5) no encephalopathy; 6) no main portal vein thrombosis; 7) no portosystemic shunts; 8) no recent gastrointestinal bleeding; 9) no Child-Pugh C.
Gerunda, Liver Transpl (2000)	1) age <70 years; 2) Child's class A or B; 3) tumor, node, metastasis (TNM) classification stages I to II (T1-T2, N0, M0); 4) patency of portal and main hepatic veins; 5) tumor size <3-5 cm and <3 nodules in the same hepatic segment; 6) no extrahepatic disease; and 7) if preoperative TACE was performed, surgery was performed only if HCC stage was confirmed at CT follow-up.	Patients living in the Veneto region underwent TACE, then surgery was performed only if their initial tumor stage was confirmed; if not, they underwent palliative treatment (TACE and alcoholization). Patients living outside the Veneto region underwent surgery immediately after standard tumor staging.
Guglielmi, HPB (2011)	NA.	NA.
Guo, Ann Surg Oncol (2014)	1) Lack of ascites; 2) hypersplenism; 3) Child-Pugh A; 4) appropriate residual liver as determined by volumetric CT.	1) Child-Pugh A; 2) lack of ascites; 3) lack of main portal vein tumor thrombus; 4) presence of hypervascular tumors on dynamic imaging.
Hasse, Langenbecks Archiv für Chirurgie (1996)	NA.	NA.
Helmberger, Digestion (2007)	Patients who had solitary HCC and normal liver function as documented by a Child-Pugh score between 5 and 6.	Patients who did not fit into the surgical criteria either for resection or liver transplantation.
Herold, Liver (2002)	No infiltration, one focus <5 cm or three foci <3 cm.	One focus <6cm or 2-5 foci <5 cm.

Ho, Ann Surg Oncol (2009)	Absolute contraindications to surgery: 1) extrahepatic metastasis; 2) tumors involving the main portal vein or inferior vena cava; 3) poor liver function (Child-Pugh C, indocyanine green retention rate at 15 min >35%; 4) bromosulphalein retention rate at 30 minutes >30%); 5) presence of other major systemic disease that may complicate the surgery.	1) Complete removal of the tumors was impossible; 2) patients refused surgical intervention; 3) tumors did not have main portal vein trunk involvement or extrahepatic metastasis; 4) serum total bilirubin concentration <2.0mg/dL.
Hsu, Eur J Radiol (2012)	1) Tumour location; 2) number of tumours; 3) Makuuchi's criteria including ascites, total serum bilirubin, indocyanine green retention rate at 15 min; 4) volumetric CT to evaluate an appropriate residual liver volume.	Hypervascular tumours on dynamic image studies.
Hsu, Ann Surg Oncol (2012)	1) Tumor invasion in single lobe or tumors in both lobes involving no more than 3 Healey's segments; 2) Child-Pugh A or B with <25% retention of indocyanine green 15min after injection; 3) no main portal vein trunk involvement or distant metastasis.	NA.
Huang, EJGH (1999)	1) Child-Pugh A or B with an indocyanine green 15 min retention rate <30%; 2) tumor involving no more than 2 Healey's segments, without portal vein main trunk involvement or distant metastasis; 3) absence of major diseases which complicate the surgery for HCC.	Refuse surgery.
Jianyong, Medicine (2014)	NA.	NA.
Jin, J Gastrointest Surg (2014)	Indications: indocyanine green 15 min retention rate <25%.<br>Contraindications: moderate to severe ascites or esophageal varix grade of ≥2.	Fear for surgical morbidity or postoperative complication.
Kang, Hepatol Int (2010)	NA.	NA.
Kirchner, Transplant Int (2011)	NA.	NA.
Lee, Hepatol Int (2014)	NA.	NA.
Lee, J Hepatol (2014)	NA.	NA.
Lin, World J Surg (2010)	1) Patients' choices after they were informed of the surgical risk and the present treatment guidelines for BCLC stage B. 2) Hepatic resection would not be performed in patients with actual residual volume by volumetric computed tomography < minimum residual liver volume.	Patients' choices.
Liu, Ann Surg Oncol (2014)	1) Patients with tumor involving no more than three Healey's segments; 2) Child-Pugh A with <25% retention of indocyanine green 15min; 3) no main portal vein trunk involvement or distant metastases.	1) Patients were not eligible or unwilling to receive curative treatments, including surgical resection, local ablation, and liver transplantation; 2) adequate liver functional reserve; 3) no signs of distant metastases or main portal trunk thrombosis.

Luo, Radiology (2011)	Treatment choice was made at the patients' request.	Treatment choice was made at the patients' request.
Markovic, J Hepatol (1998)	1) A single lesion as defined by Lipiodol CT scan; 2) liver tumors of Okuda stage I and II; 3) liver cirrhosis of Child class A and B.	1) ≥2 lesions as defined by Lipiodol CT scan; 2) Okuda stage I and II; 3) cirrhosis Child A, B and C; 4) tumor >5cm; 5) high arterial flow; 6) without marked arteriovenous shunts.
Martins, Liver Int (2006)	1) only a single lesion; 2) Child–Pugh class A; 3) Okuda stage I; 4) CLIP stage 0.	1) Single lesions >5cm; 2) well-preserved liver function; 3) portal invasion.
Min, JGH (2014)	NA.	Contraindications for TACE: 1) clinical features obviating further TACE applications; 2) occurrence of extrahepatic lesions; 3) complete portal vein thrombosis; 4) diffuse tumor growth; 5) total bilirubin >3mg/dL; 6) deteriorations to ECOG performance status ≥3; 7) Child-Pugh C.
Nagashima, Int J Oncol (1999)	NA.	NA.
Obed, Langenbecks Arch Surg (2008)	NA.	Unresectable tumours due to functional or anatomic reasons.
Park, J Gastroenterol Hepatol (2008)	NA.	NA.
Paul, Oncology (2009)	1) BCLC stage A; 2) good liver function; 3) no clinically relevant portal hypertension; 4)	1) BCLC stages B and C; 2) patent main portal vein; 3) with or without segmental or lobar portal vein invasion; 4) no extrahepatic disease; 5) BCLC stage A but not fulfilling the inclusion criteria for ablation or surgery.
Peng, Cancer (2012)	1) Generally fit; 2) resectable tumors; 3) a good hepatic reserve; 4) patients' choice.	1) Generally fit; 2) resectable tumors; 3) a good hepatic reserve; 4) patients' choice.
Perry, Liver Int (2007)	1) Child-Pugh A; 2) absence of significant portal hypertension (including hepatic venous pressure gradient in those without overt portal hypertension); 3) 3 tumours confined to one lobe.	Patients who were not suitable for resection or transplantation.
Sako, Anticancer Research (2003)	1) Serum bilirubin <2 mg/dl; 2) albumin >3.0 g/dl; 3) prothrombin time ratio >70%; 4) indocianine green retention rate at 15 minutes <20%; 5) operable lesion; 6) receipt of informed consent; 7) no co-existence of severe complications.	Not treated with surgery or ablation therapy.
Sasaki, J Hepatobiliary Pancreat Surg (1998)	NA.	NA.
Schumacher, Ann Hepatol (2010)	NA.	NA.
Sotiropoulos, Dig Dis Sci (2009)	1) Anatomically resectable disease; 2) adequate reserve liver function.	1) Patients did not qualify for liver resection; 2) no portal vein thrombosis; 3) no arterio-portal fistula; 4) no extrahepatic disease; 5) absence of decompensated liver cirrhosis.

Toro, BMC Surg (2014)	Child-Pugh class A disease; indocyanine green retention rate at 15 min, as evaluated by the Makuuchi algorithm.	1) Either Child-Pugh class A or B disease; 2) multiple tumors not suitable for surgery or radiofrequency ablation.
Ueno, J Hepatobiliary Pancreat Surg (2002)	1) Indocyanine green 15 min retention rate < 40%; 2) tumor type was not diffuse or massive.	1) Surgery was not feasible because of decreased hepatic reserve and expected liver cell loss due to resection; 2) main portal branch tumor thrombus.
Utsunomiya, Ann Surg (2014)	No detailed information were provided. The treatment algorithm is based on 3 factors: "degree of liver damage", "number of tumors," and "tumor diameter".	No detailed information were provided. The treatment algorithm is based on 3 factors: "degree of liver damage", "number of tumors," and "tumor diameter".
Wang, Academic Journal of Second Military Medical University (2012)	NA.	NA.
Wang, Dig Liver Dis (2013)	Individual decisions regarding treatment were made by the clinical physicians and their patients.	Individual decisions regarding treatment were made by the clinical physicians and their patients.
Worns, Scand J Gastroenterol (2012)	Patients were not treated by surgery: 1) the tumor was too expanded (multifocality, located on both liver lobes, extrahepatic spread); 2) comorbidity or performance status inhibited a surgical approach.	Unresectable tumors without macroscopic vascular invasion of the main portal vein or extrahepatic spread.
Yamagiwa, J Gastroenterol Hepatol (2008)	Criteria of Miyagawa et al. (Miyagawa S, et al. Criteria for safe hepatic resection. Am J Surg. 1995; 169:589–94.) Contraindication: main portal vein thrombosis, Child-Pugh C, severe arteriovenous shunting.	All stages of HCC.
Yang, Radiology (2014)	Well-preserved liver function.	Curative therapies including hepatic resection and radiofrequency ablation were not possible.
Ye, World J Gastroenterol (2014)	Moderate hepatic function (Child-Pugh A or B) and sufficient functional hepatic reserve.	Moderate hepatic function (Child-Pugh A or B) but insufficient hepatic functional reserve.
Yin, J Hepatol (2014)	See the patient selection.	See the patient selection.
Zhang, J Surg Res (2014)	1) Dominant lesion could be resected enbloc, whereas minor lesion(s) in the contralateral lobe could also be resected or ablated; 2) Child-Pugh A or B, the indocyanine green retention rate at 15 min <20% and adequate future remnant liver volume could be preserved; 3) no tumor thrombosis involved portal trunk or inferior vena cava; 4) no previous or simultaneous malignancies; 5) no extrahepatic metastasis.	1) Complete removal of the tumors was impossible; 2) patients refused surgical intervention.
Zhong, Ann Surg (2014)	1) Appropriate residual liver volume determined by volumetric computed tomography; 2) lack of hepatic encephalopathy.	Lack of main portal vein tumor thrombus.

Abbreviations: BCLC, Barcelona Clinic Liver Cancer; ECOG, Eastern Cooperative Group; HCC, hepatocellular carcinoma; NA, not available; PVTT, portal vein tumor thrombus; TACE, transarterial chemoembolization.
