# Supplementary material for: Hepatic resection versus transarterial chemoembolization for the initial treatment of hepatocellular carcinoma: A systematic review and meta-analysis
Source: Oncotarget. 2015 May 14;6(21):18715–33. doi: 10.18632/oncotarget.4134 (PMC4621923; doi:10.18632/oncotarget.4134)
Supplement: Supplementary file 5 [file oncotarget-06-18715-s005.pdf]

**Supplementary Table 4. Study quality assessment: An overview of included studies**

| First author, Journal (Year) | Q1 | Q2 | Q3 | Q4 | Q5 | Q6 | Q7 | Q8 | Q9 | Quality |
|---|---|---|---|---|---|---|---|---|---|---|
| Cheng, Zhonghua Zhong Liu Za Zhi (2005) | No | Yes | Yes | Unclear | Yes | Unclear | Unclear | No | Unclear | Low |
| Choi, World J Gastroenterol (2013) | No | No | No | Yes | No | Unclear | No | Yes | Unclear | Low |
| Ciria, J Hepatol (2014) | No | Unclear | Unclear | No | No | Yes | Unclear | Yes | Unclear | Low |
| Colella, Transpl Int (1998) | No | Unclear | Unclear | Unclear | Unclear | Unclear | Unclear | Yes | Unclear | Low |
| El-Serag, J Hepatol (2006) | Unclear | Yes | Yes | No | Unclear | Unclear | Unclear | No | Unclear | Low |
| Fan, Eur J Surg Oncol (2014) | No | Yes | Yes | Yes | Yes | Unclear | No | Yes | Unclear | Moderate |
| Gerunda, Liver Transpl (2000) | Yes | Unclear | Unclear | Unclear | Unclear | Unclear | No | Yes | Unclear | Low |
| Guglielmi, HPB (2011) | No | Unclear | Unclear | Unclear | Unclear | Unclear | Unclear | No | Unclear | Low |
| Guo, Ann Surg Oncol (2014) | No | Yes | Yes | Yes | Yes | Yes | No | Yes | Unclear | Moderate |
| Hasse, Langenbecks Archiv für Chirurgie (1996) | Unclear | Yes | Yes | Unclear | Unclear | No | Unclear | No | Unclear | Low |
| Helmberger, Digestion (2007) | No | Unclear | Yes | Unclear | Yes | Yes | No | No | Unclear | Low |
| Herold, Liver (2002) | No | Unclear | Unclear | Unclear | Unclear | Unclear | No | Yes | Unclear | Low |
| Ho, Ann Surg Oncol (2009) | No | No | Yes | No | No | Unclear | No | Yes | Unclear | Low |
| Hsu, Eur J Radiol (2012) | No | Yes | Yes | Yes | No | Yes | No | Yes | Yes | Moderate |
| Hsu, Ann Surg Oncol (2012) | No | Yes | Yes | Yes | Yes | Yes | Unclear | Yes | Unclear | Moderate |
| Huang, EJGH (1999) | Unclear | No | Yes | Unclear | Yes | Unclear | No | No | Unclear | Low |
| Jianyong, Medicine (2014) | No | Yes | Yes | Yes | Yes | Yes | Unclear | No | Yes | Moderate |
| Jin, J Gastrointest Surg (2014) | No | Yes | Yes | Yes | Yes | Yes | No | Yes | Unclear | Moderate |
| Kang, Hepatol Int (2010) | No | Unclear | Unclear | Unclear | Unclear | Unclear | Unclear | No | Unclear | Low |
| Kirchner, Transplant Int (2011) | No | Unclear | Unclear | Unclear | Unclear | Unclear | Unclear | Yes | Unclear | Low |
| Lee, Hepatol Int (2014) | Unclear | Unclear | Unclear | Unclear | Unclear | Unclear | Unclear | No | Unclear | Low |
| Lee, J Hepatol (2014) | Unclear | Unclear | Unclear | Unclear | Unclear | Unclear | Unclear | No | Unclear | Low |
| Lin, World J Surg (2010) | No | Yes | Yes | Yes | No | Yes | No | No | Yes | Moderate |
| Liu, Ann Surg Oncol (2014) | No | Yes | Yes | Yes | Yes | Yes | No | Yes | Unclear | Moderate |
| Luo, Radiology (2011) | Yes | No | Yes | Yes | Yes | Unclear | Unclear | No | Yes | Moderate |
| Markovic, J Hepatol (1998) | Yes | Unclear | Unclear | Unclear | Unclear | Unclear | No | Yes | Unclear | Low |
| Martins, Liver Int (2006) | No | Unclear | Unclear | Unclear | Unclear | Unclear | No | Yes | Unclear | Low |

| | | | | | | | | | | |
|---|---|---|---|---|---|---|---|---|---|---|
| Min, J Gastroenterol Hepatol (2014) | No | Yes | Yes | Yes | Yes | Yes | Unclear | Yes | Unclear | Moderate |
| Nagashima, Int J Oncol (1999) | No | Yes | Yes | Unclear | No | Unclear | Unclear | No | Unclear | Low |
| Obed, Langenbecks Arch Surg (2008) | No | Unclear | No | Unclear | No | Unclear | Unclear | Yes | Unclear | Low |
| Park, J Gastroenterol Hepatol (2008) | Yes | Unclear | Unclear | Unclear | Unclear | Unclear | No | Yes | Unclear | Low |
| Paul, Oncology (2009) | No | Unclear | Unclear | Unclear | Unclear | Unclear | No | Yes | Yes | Low |
| Peng, Cancer (2012) | No | Yes | Yes | Yes | No | Unclear | No | Yes | Unclear | Moderate |
| Perry, Liver Int (2007) | Yes | Unclear | Unclear | Unclear | Unclear | Unclear | No | Yes | Yes | Low |
| Sako, Anticancer Research (2003) | No | Unclear | Unclear | Unclear | Unclear | Unclear | No | Yes | Unclear | Low |
| Sasaki, J Hepatobiliary Pancreat Surg (1998) | No | Unclear | Unclear | Unclear | Unclear | Unclear | Unclear | No | Unclear | Low |
| Schumacher, Ann Hepatol (2010) | No | Yes | Yes | No | Unclear | No | Unclear | No | Unclear | Low |
| Sotiropoulos, Dig Dis Sci (2009) | No | Yes | No | Yes | No | No | No | Yes | Unclear | Low |
| Toro, BMC Surg (2014) | No | Unclear | Yes | No | Unclear | Unclear | No | No | Unclear | Low |
| Ueno, J Hepatobiliary Pancreat Surg (2002) | Yes | Yes | Yes | No | No | No | No | No | Unclear | Low |
| Utsunomiya, Ann Surg (2014) | Yes | No | No | No | No | No | No | Yes | Unclear | Low |
| Wang, Academic Journal of Second Military Medical University (2012) | Unclear | Yes | Yes | Yes | Yes | Unclear | Unclear | No | Unclear | Moderate |
| Wang, Dig Liver Dis (2013) | No | Unclear | Unclear | Yes | Unclear | Yes | Unclear | No | Unclear | Low |
| Worns, Scand J Gastroenterol (2012) | No | Unclear | Unclear | Unclear | Unclear | Unclear | No | No | Yes | Low |
| Yamagiwa, J Gastroenterol Hepatol (2008) | No | Unclear | Unclear | Unclear | Unclear | Unclear | No | Yes | Unclear | Low |
| Yang, Radiology (2014) | No | Yes | Yes | No | Yes | Yes | No | No | Unclear | Moderate |
| Ye, World J Gastroenterol (2014) | No | Yes | Yes | Yes | Yes | Unclear | No | No | Unclear | Moderate |
| Yin, J Hepatol (2014) | Yes | Yes | Yes | Yes | Yes | Yes | Yes | Yes | Yes | High |
| Zhang, J Surg Res (2014) | No | Yes | Yes | No | No | Unclear | No | Yes | Unclear | Low |
| Zhong, Ann Surg (2014) | No | Yes | Yes | Yes | Yes | Yes | No | No | Unclear | Moderate |
